# Metabolic alterations impair differentiation and effector functions of CD8+ T cells

**DOI:** 10.3389/fimmu.2022.945980

**Published:** 2022-08-02

**Authors:** Antonio Bensussen, Maria Angelica Santana, Otoniel Rodríguez-Jorge

**Affiliations:** ^1^ Laboratorio de Dinámica de Redes Genéticas, Centro de Investigación en Dinámica Celular, Universidad Autónoma del Estado de Morelos, Cuernavaca, Mexico; ^2^ Laboratorio de Inmunología, Centro de Investigación en Dinámica Celular, Universidad Autónoma del Estado de Morelos, Cuernavaca, Mexico

**Keywords:** CD8+ T cells, metabolism, ROS, mTORC2, high-fat diet, alcohol consumption, diabetes, systems biology

## Abstract

CD8+ T lymphocytes are one of the main effector cells of the immune system, they protect the organism against intracellular threats such as viruses and bacteria, as well as neoplasms. It is currently well established that CD8+ T cells have distinct immune responses, given by their phenotypes Tc1, Tc2, Tc17, and TcReg. The cellular plasticity of such phenotypes depends on the presence of different combinations of cytokines in the extracellular medium. It is known that metabolic imbalances play an important role in immune response, but the precise role of metabolic disturbances on the differentiation and function of CD8+ T cells, however, has not been explored. In this work, we used a computational model to explore the potential effect of metabolic alterations such as hyperglycemia, high alcohol consumption, dyslipidemia, and diabetes on CD8+ T cell differentiation. Our model predicts that metabolic alterations preclude the effector function of all CD8+ T cell phenotypes except for TcReg cells. It also suggests that such inhibition originates from the increase of reactive oxygen species in response to metabolic stressors. Finally, we simulated the outcome of treating metabolic-inhibited CD8+ T cells with drugs targeting key molecules such as mTORC1, mTORC2, Akt, and others. We found that overstimulation of mTORC2 may restore cell differentiation and functions of all effector phenotypes, even in diabetic patients. These findings highlight the importance of our predictive model to find potential targets to strengthen immunosuppressed patients in chronic diseases, like diabetes.

## Introduction

CD8+ T cells are the main arm of adaptive immunity against intracellular pathogens and cancer (1–3). An adequate CD8+ T cell response generates immunity against intracellular pathogens like viruses and bacteria and eradicates transformed cells, while low or deficient responses can lead to recurrent infections and cancer. It has been established that an impaired CD8+ T cell response is at the root of autoimmune diseases. Moreover, CD8+ T cells are being used or targeted for the treatment of cancer (3). Therefore, describing and modeling CD8+ T cell responses are of crucial importance for making predictions, discovering new treatments, and defining Immune therapies.

CD8+ T cells are activated when they recognize their peptide antigen, bound to MHC class I on the surface of antigen presenting cells (APC). After recognizing their antigen and receiving costimulatory signals, like those mediated by the CD28 receptor, as well as cytokine signals, naïve CD8+ T cells expand (proliferate) and differentiate into several effector and memory phenotypes to eradicate the pathogen (2). The main effector phenotype is the cytotoxic CD8+ T cell (CTL), which kills infected and neoplastic cells (3). Later, when the threat is no longer present, CD8+ T cell response contracts by means of apoptosis and only memory cells remain, conferring a lasting protection (immunological memory) against the same antigen.

CD8+ T cell differentiation leads to several functional phenotypes, with specific molecular markers and cytokine production. Up to five CD8+ T cell effector phenotypes have been widely described: Tc0, Tc1, Tc2, Tc17, TcReg (4, 5). Each of these effector cells is generated by a combination of antigen, costimulatory and cytokine signals, and carries common and specific immune functions. The integration of those complex signals is crucial for determining the differentiation path of CD8+ T cells.

For studying CD8+ T cell signal integration and differentiation, computational and mathematical approaches can be very useful. Indeed, logical modeling strategies have proved their value by allowing novel insights into the molecular dynamics of T cell activation and differentiation (6–8). Actually, several mathematical and computational modeling strategies have been used in the past for studying CD8+ T cell activation and differentiation (9–11), but the subject remains poorly explored. For instance, the role of metabolism on CD8+ T cell fate decision. The networks responsible for the generation of multiple CD8+ T cell effector phenotypes are intricate, and new experimental information is constantly generated, thus requiring actualizations and novel integrative studies.

The aim of this work is to understand the role of metabolic alterations in the differentiation and effector functions of CD8+ T cells. To this end, we used a qualitative logical modeling approach to generate a computational model that describes the core genetic and signaling network underlying CD8+ T cell immune responses. We used public experimental data to validate the model outputs, in particular CD8+ T cell functions in the context of the metabolic dysregulation present in a diabetic person. We also used our model to identify putative *knock-out* and *knock-in* targets that could potentially lead to an improved CD8+ T cell response. Finally, we validated our predictions using public experimental data. The computational model and the methodology developed here can be used to study the role of CD8+ T cells in other pathogenic scenarios. To our knowledge, this is the first computational model integrating signaling, metabolic and genetic pathways to describe the differentiation of CD8+ T cells into the main effector phenotypes.

## Materials and methods

### Construction of the gene regulatory network of CD8+ T cells

To build the network, we first reviewed the literature to extract the signaling, metabolic and genetic pathways underlying the immunological functions of CD8+ T lymphocytes (12–21), as well as that regarding gene expression of CD8+ T cell functional phenotypes (22–37). Next, we used the Human Protein Atlas (proteinatlas.org) (38) to identify which genes are expressed on CD8+ T cells, then we searched for signaling and metabolic interactions of such genes in Kyoto Encyclopedia of Genes and Genomes, KEGG (39–41), to further refine the network. We avoided the use of predicted interactions since there is not enough experimental evidence to sustain such interactions. Finally, we graphically summarized our findings in the network shown in **Supplementary File 1**.

### Derivation of computational model

**Figure 1:**
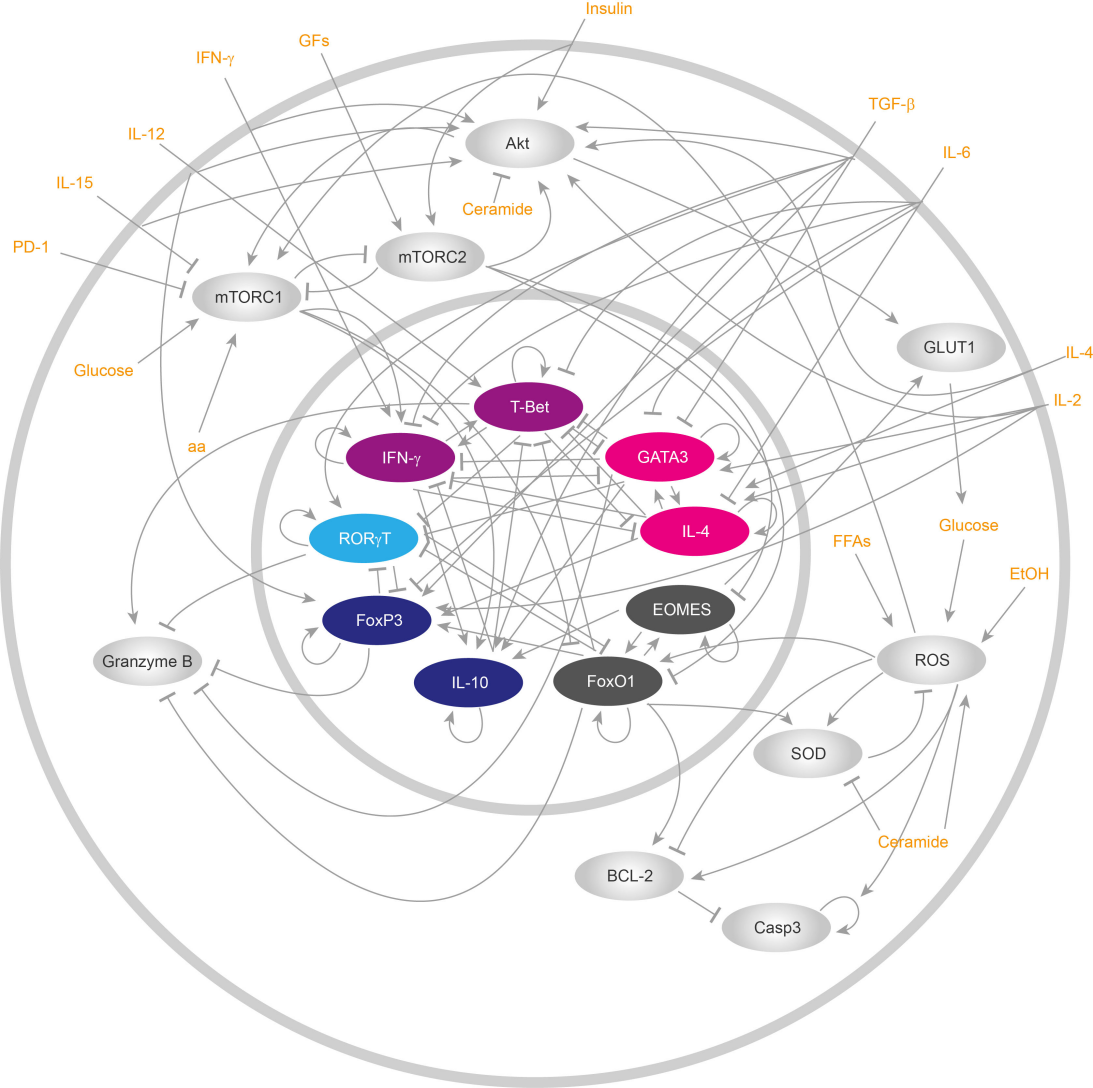
Reduced gene regulatory network of CD8+ T cells. ReducedCD8+ T lymphocyte gene regulatory network. All cytoplasmic components are drawn in silver gray, while nuclear components are represented with colors. All inputs of the networks, i.e., all external stimuli that either activate or repress the functioning of the network, are displayed in orange.

After obtaining a global overview of the main genetic regulatory, metabolic and signaling processes underlying CD8+ T cell functions, we reduced the gene regulatory network (GRN), focusing on the main non-linear motifs of the network (**Figure 1**). The network reduction procedure is detailed in **Supplementary Material**. Briefly, intermediate not self-regulated nodes were removed from the network, and direct interactions were drawn from the regulators to the targets of the removed node, while keeping positive and negative feedback loops. We then used the Boolean formalism to translate the biological interactions into logic rules associated with the main components of the simplified GRN (**Table 1** and **Supplementary Material**). In general, each logic rule assumes that every gene or any biologically relevant molecule of the network can be expressed in binary terms, i.e., “active” or “inactive”, “expressed” or “not expressed”. Numerical equivalences for these states are 1 for “activation” and 0 for “inactivation”. Then, the current state of each node of the GRN (*x*_n_) can be calculated considering the state of its regulators, which are nodes upstream that downregulate or upregulate the target node. Formally, it can be expressed as follows:


$$x_{i}\left(t+\Delta t\right)=f_{i}\left(x_{1}\left(t\right),x_{2}\left(t\right),\ldots ,x_{k}\left(t\right)\right)$$

Where *k* represents the total number of regulators for a node and *f_i_* represents a Boolean function that dictates which interactions a node has with other network components (42) (**Table 1**). The nodes of the network might present state changes at each time step, that is, they can be updated synchronously. On the other hand, a more realistic way to represent state changes of all nodes is to assign a certain time to each molecular interactions to make its own change of state, updating each node asynchronously. In this regard, we used both strategies to update the nodes in our computational model (synchronous and asynchronous).

**Table 1:** Logic rules of the CD8+ T cells gene regulatory network.

Node	Logic rule*
T-BET	((*IFN*γ ˅ (*IL*12*s* ˄¬ (*IL*6*s* ˅ *IL*4 ˅ *IL*10))) ˅ *TBET*) ˄¬ (*IL*4 ˅ *GATA*3 ˅ *IL*6*s* ˅ *F*0*X0*1)
IFN-γ	((*IFN*γ*s* ˅ *IFNI* ˅ ((*IFN*γ ˅ TBET ˅ *EOMES*) ˄ *mTORC*1˄¬ (*GATA*3 ˅ *TGFβ*))) ˄¬ *IL*6s ˅ *IL*4 ˅ *IL*10)
GATA3	((*IL*2*s* ˄ *IL*4) ˅ *EOMES* ˅ *GATA*3) ˄¬ (*TBET* ˅ *TGFβ* ˅ *IL*6s ˅ *IFN*γ
IL-4	(*IL*4s ˅ (*GATA*3 ˄ (*IL*2*s* ˅ *IL*4) ˄¬ (*TBET*)) ˄¬ (*IFN*γ ˅ *IL*6s)
RORγT	(*IL*6*s* ˄ *TGFβ* ˄¬( *TBET* ˅ *FOXP*3 ˅ *GATA*3 ˅ *FOX0*1)
IL-10	(*IL*10*s* ˅ *EOMES* ˅ (*IL*10 ˄ (*IFN*γ ˅ *IL*6s ˅ *TGFβ* ˅ *GATA*3))) ˄ *mTORC*1
FOXP3	((*IL*2*s* ˅ *IL*12*s*) ˄ *TGFβ* ˅ *FOXP*3 ˅ *IL*4 ˅ (*FOX0*1 ˄¬ (*IL*6s ˅ *ROR*γ*T*)
FOXO1	(*ROS* ˅ *FOX0*1) ˄¬( *mTORC*1 ˅ *mTORC*2)
EOMES	(*ROS* ˅ *EOMES* ˅ *IFN1* ˄ *FOX0*1 ˄¬( *mTORC*2)
mTORC1	(*aa* ˅ *ROS* ˅ *IL1*2 ˅ *IL1*2*s* ˅ *Akt*) ˄¬ (*mTORC*2 ˄ *PD*1 ˄ *IL*15s)
mTORC2	(*GF*s ˅ *ROS*) ˄ ¬*mTORC*1
ROS	(*Glucose* ˅ *FFAs* ˅ *Ceramide* ˅ *Et0H*) ˄¬*SOD*
Akt	(*IFN*γ ˅ *IL*4 ˅ *IL*10 ˅ *mTORC*2)
GLUT1	*Akt* ˄ *EOMES* ˄ ¬*FOX0*1
Granzyme B	*TBET* ˄ ¬(*GATA*3 ˅ *ROR*γ*T* ˅ *FOXP3* ˅ *FOX01*)
SOD	*ROS* ˄ (*FOX0*1) ˄ ¬(*Ceramide*)
BCL-2	(*FOX*01) ˄ ¬*ROS*
Casp-3	*FasL* ˄(*ROS* ˅ *Casp*3) ˄ ¬*BCL*2

*In this network IL12s, IL6s, TGF-β, IL4s, IL10s, IFN-γs, IFNI, Glucose, FFAs, Ceramide, FasL and EtOH are inputs. This means that such molecules are considered as external stimuli of CD8+ T cells. In this table we used ˄to represent the logic operator “AND”, ˅ to represent “OR” and finally “¬ “ to represent “NOT”.

### Implementation of Boolean models

We implemented each update strategy in Microsoft Visual Studio 2022 using the C# programming language. For the synchronous updating strategy, we updated all nodes at every time step. On the other hand, for implementing the asynchronous updating strategy we assigned a specific updating time for each node, which was determined considering the type of nodes, i.e., whether the nodes correspond to metabolites, signaling components, RNAs, or newly expressed proteins. Also, we considered whether the nodes were implicated on transcriptional cascades or not to reevaluate the duration of the updating time. In **Supplementary Table 1**, we present all updating times for the asynchronous case. The algorithm to implement synchronous and asynchronous strategies first calculates all possible initial conditions of the network, given by the space of configurations *Ω=*2*^n^*, where “*n*” is the total number of nodes. Then, the algorithm picks one of the initial configurations from Ω, and initiates the simulation using the logic rules presented in **Table 1**. The individual simulation ends when a fixed attractor is found (stable state). Attractors of the type “fixed point” or “stable state”, in biological terms represent stable genotypes that can be grouped in phenotypes, depending on the state of certain markers used to define the phenotype.

We used labeling logic rules to identify each fixed attractor (i.e. genotype) with their corresponding phenotype, as it has been done for other biological networks such as in CD4+ T cells (43). All labelling rules were constructed and enriched with data from experimental literature and are presented in **Table 2**. We included such identifier rules in our algorithm to classify the attractors found, and we systematically used all configurations contained in Ω. In this way, we obtained all attractors of the network, as well as the number of configurations that reach each attractor (i.e. the size of its basin of attraction).

**Table 2:** Phenotype identifier.

Node	Logic rule
Naïve	¬(*EOMES* ˅ *TBET* ˅ *GATA*3 ˅ *ROR* γ*T* ˅ *FOXP*3)
Effector	¬*FOX0*1 ˄ *EOMES* ˄ (*TBET* ˅ GATA3 ˅ *ROR* γ*T* ˅ *FOXP*3)
Memory	*FOX0*1 ˄ *EOMES* ˄ (*TBET* ˅ *GATA*3 ˅ *ROR*γ*T* ˅ *FOXP*3 )
Tc0	¬(*EOMES* ˅ *TBET* ˅ *GATA*3 ˅ *ROR*γ*T* ˅ *FOXP*3)
Tc1	*EOMES* ˄ (*TBET* ˄ *IFN*γ) ˄ ¬ *FOX0*1 ˄ ¬ (*GATA*3 ˅ *ROR*γ*T* ˅ *FOXP*3)
Tc2	*EOMES* ˄ (*GATA*3 ˄ *IL*4) ˄ ¬ *FOX0*1 ˄ ¬(*TBET* ˅ *ROR*γ*T* ˅ *FOXP*3)
Tc17	*EOMES* ˄ (*ROR*γ*T*) ˄ ¬ *FOX0*1 ˄ ¬(*TBET* ˅ *GATA*3 ˅ *FOXP*3)
TcReg	*EOMES* ˄ (*FOX03* ˄ *IL*10) ˄ ¬ *FOX0*1 ˄ ¬(*TBET* ˅ *GATA*3 ˅ *ROR*γ*T*)

In this table we used ˄to represent the logic operator “AND”, ˅ to represent “OR” and finally “¬ “ to represent “NOT”.

### Dynamical simulations of the models

Previous publications showed that the size of the basin of attraction is a useful qualitative tool to determine the prevalence of a certain phenotype (44). Thus, we used the size of the basin of attraction to estimate the relative frequency of a phenotype inside a cell population. To calculate the frequency of each CD8+ T cell phenotype, we used the following equation:


$$f_{k}=\frac{y_{k}}{\Omega }\times 100\%$$

Where *f_k_* is the frequency of the “*k*” phenotype of CD8+ T cells, *y_k_* is the total amount of initial configurations that reach attractors (stable states) of the “*k*” phenotype and Ω is the size of the entire states space of the GRN. Furthermore, we perturbed the GRN by performing knock-out and knock-in simulations to estimate the effect of changes on specific nodes in the dynamics of the model and their contribution to specific phenotypes. In this sense, all knockouts were simulated by setting *x*_i_(*t* + *Δt*)=0 for the target gene, and similarly, all knock-ins were simulated by *x*_i_(*t* + *Δt*)=1 for the target gene.

### Data extraction

In order to validate our model, we searched for experimental datasets obtained from flow cytometry of CD8+ T cells under particular stimulation. For instance, CD8+ T cells grown under IL-12 and IL-4 treatment. We then took the reported percentage of cells that correspond to phenotypes Tc0, Tc1, Tc2, Tc17 and TcReg, which is calculated by dividing the total count of cells that were positive to a specific gene marker (IFN-γ+ for Tc1, IL-4+ for Tc2, RORγT+ for Tc17 and FoxP3+ for TcReg cells) by the total count of living cells in the sample. We only used experimental sources that directly report such percentages, without using third-party software for data extraction. All sources are detailed forward.

### Model availability

The source code for this work is freely available at:

https://github.com/ABensussen/CD8-T-cells/tree/main.

The Ginsim implementation of the model used to compute all model attractors is presented as **Supplementary File 1**.

## Results

### A computational model that reproduces *in vitro* observations of phenotypic differentiation of CD8+ T cells

**Figure 2:**
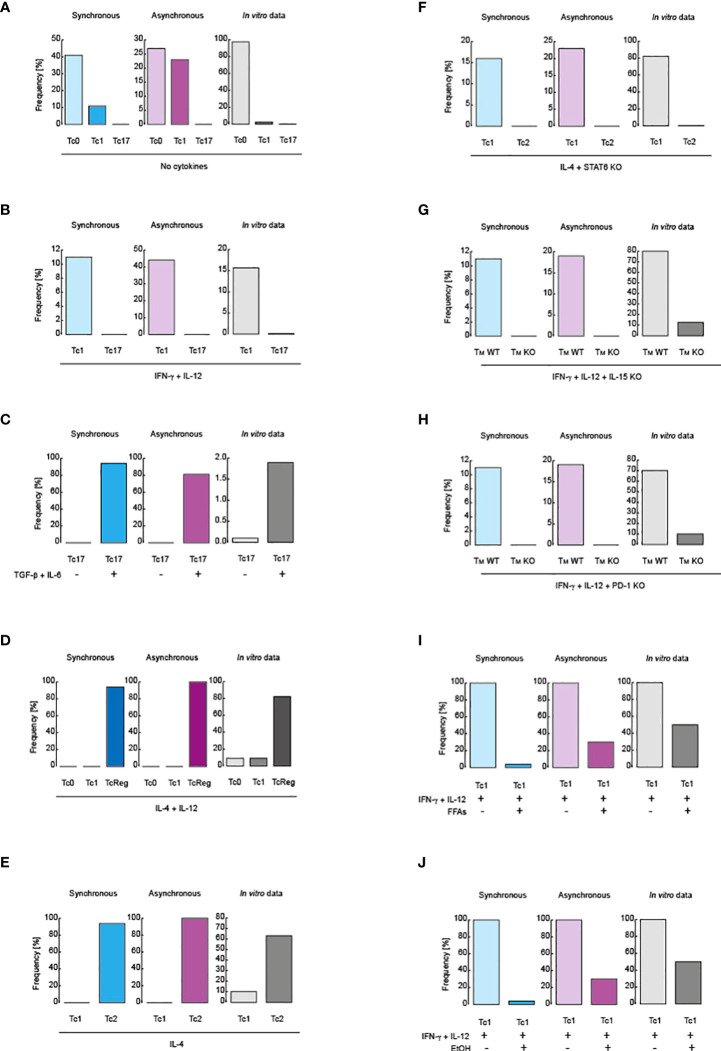
Validation of the models. Comparison between simulated data and flow cytometry data obtained from *in vitro* assays. Blue scale of colors is assigned to the synchronous updating scheme, violet scale corresponds to the asynchronous scheme and gray scale is reserved for *in vitro* data. **(A)** Cell distribution in the absence of polarizing cytokines. **(B)** Cell distribution in the presence of IL-12 and IFN-γ. **(C)** Tc17 phenotype in before and after treatment with IL-6 and TGF-β. **(D)** Cell distribution in the presence of IL-4 and IL-12. **(E)** Cell distribution in the presence of IL-4. **(F)** Phenotype distribution in the presence of IL-4 in knockout for STAT6. **(G)** Inhibition of IL-15 signaling during IL-12 and IFN-γ stimulation. **(H)** Inhibition of PD-1 signaling in during IL-12 and IFN-γ stimulation. **(I)** Effect of FFAs during IL-12 and IFN-γ stimulation. **(J)** Effect of alcohol over IL-12 and IFN-γ stimulation. All simulations were performed in presence of IL-15, except panel **(G)**.

Scientific literature and databases curation allowed us to construct a directed GRN including 300 nodes that represents well known intracellular pathways underlying the functioning of CD8+ T cells (**Supplementary File 2**): TCR signaling, costimulatory cytokine signaling, a metabolic regulatory module, metabolic pathways, and the genetic regulatory network controlling cell fate decisions. Next, we simplified the resulting network to obtain a reduced GRN including 18 nodes that summarizes the main aspects of CD8+ T cell differentiation (**Supplementary Material**), including its connection with metabolism, such as the glycolytic pathway (**Figure 1**). Particularly, we focused this work in long term alterations of metabolism that influence CD8+ T cell differentiation. Using this approach, we were able to simplify several non-linear motives present under metabolic and signaling regulation which occur during a short time window, including the localization of some cellular components. Then, we assigned a logical rule to each node to obtain a dynamical model. The experimental evidence of the model (**Table 1**) is presented in the **Supplementary Material**. Later, we investigated whether our model represents the biology of CD8+ T cells by comparing the data obtained by computational simulations with *in vitro* observations (**Figure 2** and **Supplementary Figures 1-5**). For this comparison, we simulated the behavior of an activated CD8+ T cell population under different cytokine stimulations, and we then searched for published flow cytometry data obtained under the same conditions. We then compared the simulated cell phenotypes with the phenotypic distribution observed *in vitro*.

Regarding CD8+ T cell differentiation, it has been reported that, in the absence of cytokine stimulation, an activated population of CD8+ T lymphocytes mainly preserves the phenotype Tc0, which then generates basal levels of the Tc1 phenotype, and finally gives rise to the Tc17 phenotype (45). Both synchronous and asynchronous updating schemes qualitatively reproduced these experimental observations as shown in **Figure 2A**. In presence of IL-12 and IFN-γ, the Tc1 phenotype increases compared to the Tc17 phenotype (45), (**Figure 2B**). The Tc17 phenotype increases when the population is stimulated with IL-6 and TGF-β (45), as shown in **Figure 2C**. On the other hand, it has been reported that IL-4 with IL-12 mainly produce CD8+ T regulatory cells (TcReg) (46),, as recapitulated by our model in **Figure 2D**. Adding IL-4 alone polarizes CD8+ T cells to Tc2 phenotype, characterized by secretion of IL-4 (47) (**Figure 2E**). Importantly, these predictions are supported by the fact that the 100% of the state space converge to such phenotypes (*f*_k_ = 100%)) (**Supplementary Figures 1–5**), corresponding to stable states of the system, which ensures that our model fully explored all possible states of the GRN of CD8+ T cells.

To further test our model, we have simulated reported mutations. In this sense, it has been reported that in absence of STAT6 during stimulation with IL-4, CD8+ T cells are polarized towards the Tc1 phenotype instead of Tc2 (47), a fact that is qualitatively reproduced by our model when we suppress all nodes activated in response to STAT6 (**Figure 2F**). Moreover, it has been reported that, as a result of IL-15 signaling absence (48), during a pro-inflammatory context mediated by IFN-γ and IL-12, the generation of memory cells is minimized (**Figure 2G**). The same was also reported during stimulation with IFN-γ and IL-12 in the absence of PD-1 signaling (30) (**Figure 2H**). Furthermore, it has been reported that metabolic alterations directly affect CD8+ T cell differentiation. More precisely, it was found that during pro-inflammatory stimulation with IFN-γ and IL-12, the presence of free fatty acids (FFAs) downregulates the differentiation of Tc1 phenotype (49), which is also recapitulated by our model, as showed in **Figure 2I**. Finally, it has been observed that chronic consumption of alcohol reduces CD8+ T cell memory (50), a fact that was corroborated when we simulated a pro-inflammatory context given by IFN-γ and IL-12 in presence of ethanol, as observed in **Figure 2J**. Interestingly, the 100% of configurations contained in the state space converge to all phenotypes observed in the mutants presented in this section (i.e. *f*_k_ = 100%), which means that the model was able to explore all possible genetic combinations presented in the GRN of CD8+ T cells. Collectively, these observations show that our model accurately describes the main aspects of CD8+ T cell biology.

### Alcohol, high fat diet and metabolic imbalances impair the CD8+ T cell response

**Figure 3:**
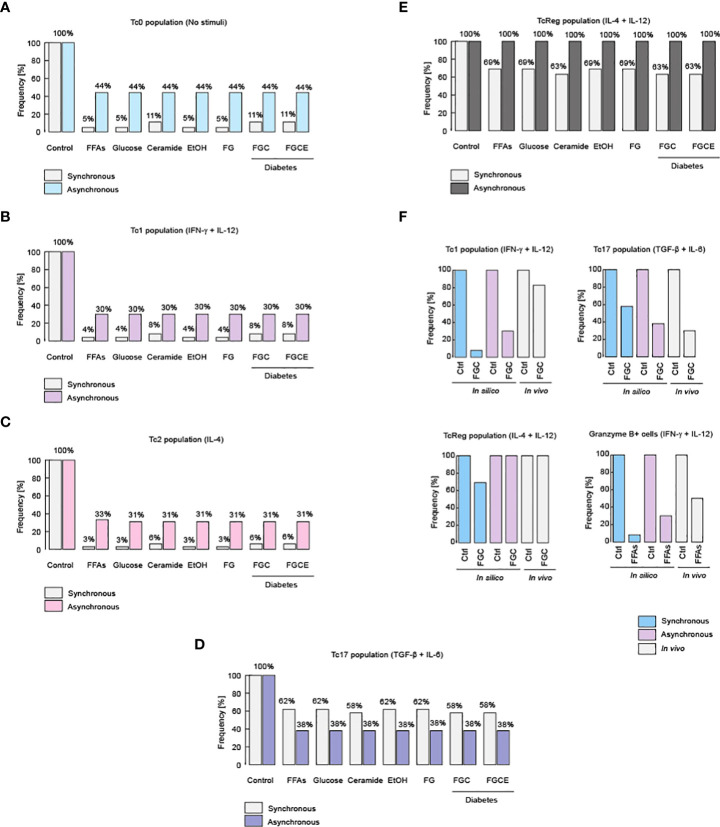
Metabolic alterations impair CD8+ T cell differentiation. Effects of different metabolic alterations such as high fat diets, high consumption of sugar and alcohol, as well as other health problems like diabetes. Metabolic alterations for the Tc0 population **(A)**, as well as for the Tc1 **(B)**, Tc2 **(C)**, Tc17 **(D)** and TcReg population **(E)**. **(F)** Experimental evidences that confirm these phenotypic alterations for Tc1, Tc17 and TcReg cells, as were described for healthy and diabetic patients (51).

Previous reports have shown that alcohol and FFAs affect the differentiation of CD8+ T cells (49, 50). Expanding such results, we investigated how nutritional imbalances affect the phenotypic differentiation of CD8+ T cells. We simulated nutritional abnormalities such as hyperglycemia, dyslipidemia, systemic high levels of ceramides and alcohol, alone or in combinations with CD8+ T cell differentiation (**Figure 3**). The combination of hyperglycemia with dyslipidemia and high levels of systemic ceramides correspond to a diabetic context (52). Both models showed that either hyperglycemia, dyslipidemia, high levels of ceramides or alcohol were able to abrogate the differentiation of CD8+ T cells to Tc0, Tc1, Tc2 and Tc17 phenotypes (**Figures 3A-D**). These results are in agreement with the fact that unhealthy nutritional habits predispose to recurrent illnesses (53).

### Regulatory CD8+ T cells are resistant to metabolic deregulation

On the other hand, our model predicts that TcReg cells are resistant to nutritional impairment, such as hyperglycemia, dyslipidemia, or high levels of ceramides and ethanol (**Figure 3E**). Contextualizing this finding, it was recently reported that TcReg cells are essential to protect against autoimmune diseases such as type I diabetes and patients with such illness have fewer TcReg counts compared to healthy individuals (12). In this respect, it was reported that the increase of intestinal microbiota produced by a diet rich in carbohydrates such as trehalose, which is a disaccharide composed by two monomers of D-glucose, stimulates TcReg proliferation (12). This implies *de facto*, that the increase in the consumption of sugars does not affect the TcReg cells, as we show with synchronous and asynchronous updating strategies (**Figure 3E**). Furthermore, the robust persistence of the TcReg phenotype may explain why autoimmune diseases such as type I diabetes have such a low frequency in the world (54). Regarding the veracity of these predictions about changes on phenotypes of CD8+ T cells, it has been reported that obese diabetic patients have reduced counts of Tc1 and Tc17 phenotypes compared to lean non-diabetic patients (51), shown by our models in **Figure 3F**. Moreover, in the same report, it was observed that TcReg cells do not present variations among diabetic and non-diabetic patients (51), a fact that demonstrates our predictions about TcReg phenotype robustness (**Figure 3F**). Furthermore, our model also predicts that during anti-tumor inflammation (i.e. stimulation of IL-12 and IFN-γ), high levels of fat reduce the population of Granzyme B-producing Tc1 cells, as recently reported (55) (**Figure 3F**). Collectively, these data suggest that dyslipidemia affects the functioning of all effector CD8+ T cells, except TcReg.

### Metabolic imbalances increase ROS and downregulate mTORC2

**Figure 4:**
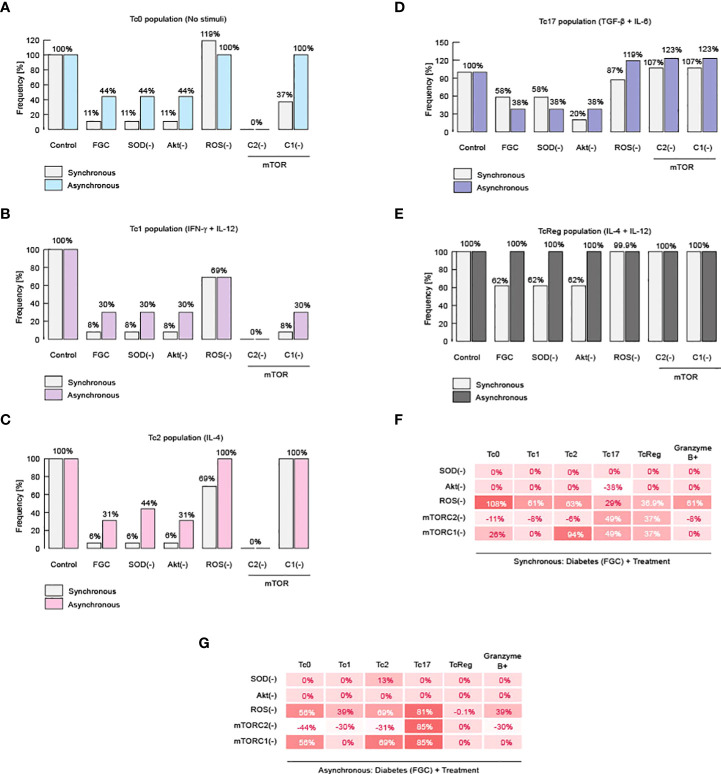
Impairment of CD8+ T cell differentiation is caused by ROS. Results of knockout simulation analysis. **(A)** Effects of knockouts over Tc0 phenotype. **(B)** Effects of knockouts over Tc1 phenotype. **(C)** Effects of knockouts over Tc2 phenotype. **(D)** Effects of knockouts over Tc17 phenotype. **(E)** Effects of knockouts over TcReg phenotype. **(F)** Summary of total changes due to knockouts according to the synchronous model. **(G)** Summary of total changes due to knockouts according to the asynchronous model.

We then wanted to determine the molecular mechanism that generates the impaired differentiation of CD8+ T lymphocytes. To answer this question, we simulated a series of knockouts directed against the regulatory module of metabolism, which comprises the nodes of superoxide dismutase (SOD), Akt, reactive oxygen species (ROS), Mammalian Target of Rapamycin Complex 1 (mTORC1) and 2 (mTORC2). We simulated such knockouts for patients that have severe metabolic alterations, whose physiological markers are hyperglycemia, dyslipidemia, and high levels of ceramide, like in type II diabetes (**Figure 4**). We observed that suppressing ROS was associated with an improvement in the generation of CD8+ T cell effector phenotypes (**Figures 4A-E**). These observations are congruent with a previous result in which the increase in ROS levels was associated to deficiencies on CD8+ T cell activation (11). Furthermore, our simulations expand the last result, because they pointed out that ROS also interfere with differentiation to Tc1, Tc2 and Tc17 effector phenotypes.

On the other hand, total abrogation of such effector phenotypes was observed when we inhibited mTORC2 (**Figures 4A-E**). These data suggest that mTORC2 is necessary to activate the immune response of CD8+ T cells (**Figures 4F,G**), a fact that has already been reported *in vitro* (56). Evidence for a link between ROS and mTORC2 was reported using *C. elegans*, in which it was observed that mTORC2 activity reduces ROS generation, enhancing cell surviving (57). Similarly, inhibition of mTORC2 in human cancerous cells increases damage produced by ROS (58). Our model indicates that this connection might be valid also in CD8+ T lymphocytes. Therefore, our results suggest that metabolic alterations increase ROS, and this impairs effector response of CD8+ T cells as well as differentiation.

### Differentiation of CD8+ T cells could be enhanced by upregulating mTORC2 activity

**Figure 5:**
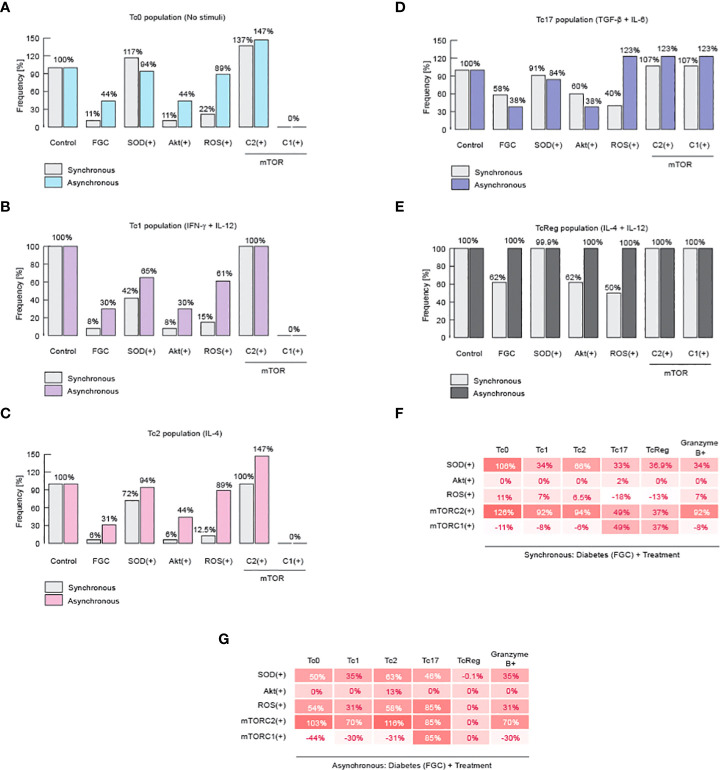
mTORC2 and inhibition of ROS might normalize metabolic alterations on CD8+ T cells. Results of knock-in simulation analysis. **(A)** Effects of knock-ins over Tc0 phenotype. **(B)** Effects of knock-ins over Tc1 phenotype. **(C)** Effects of knock-ins over Tc2 phenotype. **(D)** Effects of knock-ins over Tc17 phenotype. **(E)** Effects of knock-ins over TcReg phenotype. **(F)** Summary of total changes due to knock-ins according to the synchronous model. **(G)** Summary of total changes due to knock-ins according to the asynchronous model. FGC: diabetic context given by dyslipidemia, hyperglycemia, and high levels of ceramide.

Finally, we investigated how to reverse the damage caused by metabolic dysregulation and, similarly, we looked for more evidence to delineate the role of ROS and mTORC2 on the differentiation of CD8+ T cells. In order to achieve these goals, we perform a knock-in analysis, in which we over-expressed SOD, Akt, ROS, mTORC1 or mTORC2. We performed this analysis in the context of diabetic patients, characterized by hyperglycemia, dyslipidemia, and high systemic levels of ceramides (**Figure 5**). We found that over-expression of mTORC2 restores all effector phenotypes of CD8+ T cells (**Figures 5A-E**). Moreover, we also confirmed our initial statement about ROS, because over-expression of SOD was able to partially restore the presence of effector phenotypes (**Figures 5A-E**). Unexpectedly, we found that over-production of ROS is not completely able to restore effector phenotypes, except for Tc17 (**Figures 5B-D**).

Noteworthy, it has been previously reported that an increase in ROS is needed to activate effector CD8+ T cells through TCR signaling, but on the other hand, high and sustained levels of ROS reduce Tc1 cells and produces TcReg cells instead (59). This mechanism may explain why overproduction of ROS may partially restore all effector phenotypes during metabolic abnormalities. These results reinforce our hypothesis that mTORC2 activity is essential to maintain healthy CD8+ T effector proportions, because it fine-tunes the control over ROS levels to promote a specific immune response against micro-environment given by the cytokine milieu (**Figures 5F,G**). Therefore, metabolic abnormalities produced either by high fat diet, or high sugar diet, as well as chronic consumption of alcohol, diabetes or combination of the former possibilities may impair the effector differentiation of CD8+ T cells.

## Discussion

CD8+ T cell activation is achieved by TCR ligation, costimulatory receptors, and cytokine signals. These pathways can trigger metabolic reprogramming, especially costimulatory receptors, to make up for the energetic and precursors requirements during activation and differentiation into effector or memory phenotypes. The integration of these pathways is essential for the cell to achieve a proper response and function. Here, we presented a GRN connecting all the main pathways: TCR signaling, costimulatory and cytokines signaling, a metabolic regulatory module, metabolic pathways, and the genetic regulatory network controlling cell fate decisions. This integrative network (**Supplementary File 1**) comprises 300 nodes and was created from literature curation and database mining. From this graphical framework, we generated a concise network involving 18 nodes (**Figure 1**) and we assigned the Boolean rules to each of these nodes (**Table 1**). Our concise model was fine-tuned to recapitulate relevant experimental published data. We then simulated CD8+ T cell behavior under TCR activation, in the presence of polarizing cytokines for Tc0, Tc1, Tc2, Tc17 and TcReg, demonstrating that our model recapitulates the differentiation of CD8+ T cells into the corresponding effector (Tc0, Tc1, Tc2, Tc17 and TcReg) and memory (TM) phenotypes. Our model further account for the effects of STAT6 and IL-15 knock-outs.

Considering the importance of nutrition and metabolism for the immune system, we explored the effects of specific diet imbalances and metabolic alterations in CD8+ T responses. For this, we simulated nutritional abnormalities such as hyperglycemia, dyslipidemia, systemic high levels of ceramides and alcohol, and a diabetic context (52), with or without polarizing cytokines for CD8+ T cell differentiation (**Figures 3** and **Figure 4**). The model showed that specific components of the diet can affect the differentiation of CD8+ T cells, for instance: hyperglycemia, dyslipidemia, systemic high levels of ceramides and alcohol impair Tc0, Tc1, Tc2 and TC17 differentiation (**Figures 3A-D**). These results corroborate the fact that unhealthy nutritional habits predispose to recurrent illnesses (53). In contrast, we found that TcReg cells were resistant to nutritional impairment, such as hyperglycemia, dyslipidemia, or high levels of ceramides and ethanol (**Figure 3E**), a prediction that should be experimentally validated and furthered explored.

We then explored the role of specific nodes of the metabolic regulatory module, which comprises superoxide dismutase (SOD), Akt, reactive oxygen species (ROS), mTORC1 and mTORC2. All of these components turned out to be important for CD8 T cell differentiation, while mTORC2 proved to be essential for Tc0, Tc1 and Tc2 phenotypes, as shown by our simulation of knockouts in **Figure 4**. This result corroborate previous reports (56), in particular that Akt/mTOR pathway triggered by costimulatory receptors supports metabolic reprogramming and fulfillment of energetic requirements for T cell activation and differentiation. We also, previously reported that ROS generation and metabolic imbalances interfere with CD8 T cell functions (11).

**Figure 6:**
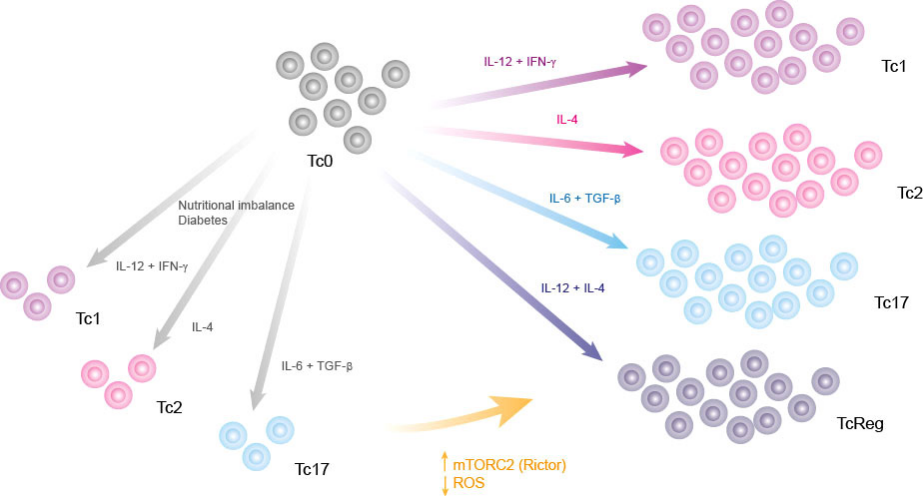
Graphical summary. Nutritional imbalances such as chronic consumption of alcohol, high fat diets, diabetes and high consumption of sugar impair the differentiation of CD8+ T lymphocytes. This deregulation of effector phenotype might be caused by an increase of ROS, which can be neutralized by targeting its production, directly with SOD stimulators or indirectly by activating mTORC2.

Once we established the relevance of mTORC2 for all functional phenotypes, we performed a *knock-in* analysis to simulate the effect of each regulatory node on CD8+ T cell function in the context of a diabetic person. We found that over-expression of mTORC2 restores all effector phenotypes of CD8+ T cells (**Figure 5**), while SOD overexpression (ROS down-regulation) was able to partially restore the effector phenotypes (**Figures 5A-E**). Unexpectedly, we found that over-production of ROS may partially restore all effector phenotypes (**Figures 5C,B,D**). This agrees with previous reports showing that ROS production is necessary for CD8+ T cell activation by TCR/CD28 signaling, while high and sustained levels of ROS reduce Tc1 differentiation favoring TcReg phenotype generation (59). We propose that metabolic alterations inhibit mTORC2 functioning, which increases the ROS production inside CD8+ T cells, leading to a reduced CD8+ T cell differentiation into effector phenotypes (**Figure 6**).

Although we found a strong correlation between the output of the model and the biological data, we should keep in mind that a possible caveat of the present work is that by reducing metabolic modules, some important interactions might have not been considered. This work, however, is focused on long-term metabolic interactions, and mainly on a metabolic regulatory module, responsible for the metabolic reprograming of effector phenotypes (Akt, mTORC1, mTORC2, ROS and SOD), which also controls the differentiation process of CD8+ T cells. The effect of specific metabolic pathways on the differentiation and functions of CD8+ T cells is a relevant topic nowadays and should be further explored.

To our knowledge, this is the first integrative mechanistic model exploring CD8+ T cell differentiation, showing the effects of an imbalanced diet and metabolic alterations in this process. Based on the analysis performed with our model, we propose an intervention for diabetic patients targeting an increase in mTORC2 activation, which can potentially restore CD8+ T cell phenotypes and function.

## Data availability statement

The original contributions presented in the study are publicly available. This data can be found here: https://github.com/ABensussen/CD8-T-cells/tree/main.

## Author contributions

AB and OR-J designed the study and wrote the first version of the manuscript. The model was generated by AB with inputs from OR-J. Model analyses were performed by AB and revised by all authors, while the Ginsim implementation was performed by OR-J. All authors contributed to the article and approved the submitted version.

## Funding

OR-J received funding from CONACYT for the realization of this work (project CF 2019 1727995).

## Acknowledgments

The authors want to thank Denis Thieffry for his generous advice and for the revision of the manuscript. AB thanks CONACYT for his postdoctoral fellowship. OR-J thanks CONACYT for the support of the project CF 2019 1727995.

## Conflict of interest

The authors declare that the research was conducted in the absence of any commercial or financial relationships that could be construed as a potential conflict of interest.

## Publisher’s note

All claims expressed in this article are solely those of the authors and do not necessarily represent those of their affiliated organizations, or those of the publisher, the editors and the reviewers. Any product that may be evaluated in this article, or claim that may be made by its manufacturer, is not guaranteed or endorsed by the publisher.
