# Supplementary material for: Metabolic alterations impair differentiation and effector functions of CD8+ T cells
Source: Front Immunol. 2022 Aug 2;13:945980. doi: 10.3389/fimmu.2022.945980 (PMC9380903; doi:10.3389/fimmu.2022.945980)
Supplement: Supplementary file 1 [file DataSheet_1.docx]

Supplementary Material

Metabolic alterations impair differentiation and effector functions of CD8+ T cells

**Antonio Bensussen^1^, Angélica Santana^2^, Otoniel Rodríguez-Jorge^2^***

^1^Laboratorio de Dinámica de Redes Genéticas, Centro de Investigación en Dinámica Celular, Universidad Autónoma del Estado de Morelos, Cuernavaca, México.

^2^Laboratorio de Inmunología, Centro de Investigación en Dinámica Celular, Universidad Autónoma del Estado de Morelos, Cuernavaca, México.

*** Correspondence:**ORJ: [orj@uaem.mx](mailto:orj@uaem.mx)

CONTENT:

[1. Biological bases of logic rules 2](#_Toc105972465)

[2. Biological bases of labeling rules 6](#_Toc105972466)

[3. Algorithm to reduce networks 8](#_Toc105972467)

[4. Stability test of phenotypes 10](#_Toc105972468)

[5. Supplementary Tables 11](#_Toc105972469)

[6. Supplementary Figures 13](#_Toc105972470)

[7. Supplementary References 19](#_Toc105972471)

## 1. Biological bases of logic rules

In this work, we collected a wide amount of data from experimental sources, and we summarized these data in Supplementary file 1. Then, we reduced this directed genetic regulatory network (GRN) to a compact form presented in Figure 1. Now, we present biological bases of each logic rule used in our model of CD8+ T cells.

**T-BET:**

According to experimental information, IL-12 activates T-BET (1), and IFN-γ (2), through STAT4. Once activated, T-BET is able to induce its own transcription in a positive feedback loop (3). Regarding negative regulators, it has been documented that IL-4 activates STAT6 (4), which in turn inhibits T-BET (5). Also, IL-6 and IL-10 activate STAT3, and this results in SOCS1 activation which is a potent inhibitor of T-BET (6). On the other hand, GATA3 is able to directly inhibit T-BET (3) as well as FOXO1 (7). All these interactions can be expressed as:

$$((IFN\gamma\vee(IL12s \wedge\neg(IL6s \vee IL4 \vee IL10)) ) \vee TBET) \wedge\neg(IL4 \vee GATA3 \vee IL6s \vee FOXO1)$$

**IFN-γ:**

Concerning IFN-γ, experimental evidence showed that type I IFN activates STAT1, which is indispensable to sustain the functioning of IFN-γ signaling (8). In the same way, IL-12 (3) and mTOCR1 (9) signaling pathways are able to activate IFN-γ expression. On the other hand, EOMES (10), T-BET, and IFN-γ (3) form positive feedback loops to sustain expression of the later. Nevertheless, other important molecules are able to abrogate such sustained loop of activation. In this sense, IL-6 and IL-10 through STAT3 activate SOCS1, which inhibits IFN-γ (6). Similarly, IL-4 also activates SOCS1, turning off IFN-γ (11). At the transcriptional level, GATA3 (12), FOXO1 (13) and TGF-β (14) inhibit IFN-γ. Thus, this information can be expressed as follows:

$$(IFN\gamma s \vee IFNI \vee((IFN\gamma\vee TBET \vee EOMES) \wedge mTORC1 \wedge\neg(GATA3 \vee TGF\beta))) \wedge\neg(IL6s \vee IL4 \vee IL10)$$

**GATA3:**

Regarding GATA3, it has been observed that IL-2, IL-4 and IL-13 induce GATA3 expression (15), and once activated, this transcription factor is able to activate its own expression (16). Transcription of GATA3 may be activated by EOMES (17) and in opposition to this interaction, T-BET (3) represses GATA3. At signaling level, IL-6 activates SOCS1, which is a potent inhibitor of GATA3 (6). In the same way, IFN-γ inhibits GATA3 by promoting T-BET expression (3). Thus, summarizing this information:

$$\left( \left( IL2s \wedge IL4 \right)\vee EOMES \vee GATA3 \right)\wedge\neg\left( TBET \vee TGF\beta\vee IL6s \vee IFN\gamma\right)$$

**IL-4:**

About regulation of IL-4, it has been reported that IL-2 signaling is able to activate IL-4 gene expression (11), as IL-4 itself does (18). At transcriptional level, GATA3 activates IL-4 expression (16). On the contrary, T-BET and IFN-γ inhibit IL-4 expression (19) and similarly, IL-6 signaling inhibit IL-4 through enhancing the SOCS1 activity (6). Collectively, this can be represented as follows:

$$(IL4s \vee(GATA3 \wedge(IL2s \vee IL4) \wedge\neg TBET)) \wedge\neg(IFN\gamma\vee IL6s )$$

**RORγT:**

It has been documented that IL-6 and TGF-β selectively activate RORγT (20), while other transcription factors such as FOXO1 (21), FOXP3 (22), GATA3 (23) and T-BET (24) inhibit RORγT. In other words:

$$(IL6s \wedge TGF\beta) \wedge\neg(TBET \vee FOXP3 \vee GATA3 \vee FOXO1)$$

**IL-10:**

It has been reported that IL-10 gene regulation in CD8+ T cells has especial features, for instance, in this cells IFN-γ activate expression of IL-10 (25–27). Also, other important cytokines such as IL-6 and IL-10 can activate STAT3 (6), and this transcription factor induce IL-10 gene expression (28). On the other hand, TGF-β activates IL-10 expression in a STAT3-independent manner (29). At transcriptional level, EOMES (30) and GATA3(31) activates IL-10 expression. The last interaction to consider is that mTORC1 increases IL-10 (32). Therefore, these data can be summarized as follows:

$$(IL10s \vee EOMES \vee(IL10 \wedge(IFN\gamma\vee IL6s \vee TGF\beta\vee GATA3 ))) \wedge mTORC1$$

**FOXP3:**

Regarding FOXP3, it has been demonstrated that IL-2 (33), IL-4 (34) and IL-12 (26) activate FOXP3 in CD8+ T cells. Once active, FOXP3 starts a sustained positive feedback loop that maintains its levels inside cells (35). This loop can be interrupted by IL-6 (20) and TGF-β (35) signaling. At transcriptional level, RORγT (20) and FOXO1 (35) inhibit FOXP3 self-activation. Thus, these data may be summarized as follows:

$$((IL2s \vee IL12s) \wedge(TGF\beta\vee FOXP3 \vee IL4 \vee(FOXO1))) \wedge\neg(IL6s \vee ROR\gamma T )$$

**FOXO1:**

Concerning FOXO1, this transcription factor is able to produce a positive feedback loop that sustains its own gene expression (36). However, metabolic changes such as the presence of ROS (37) may increase the expression of FOXO1. On the contrary, mTOR1 signaling represses FOXO1 function (7). Then, using this information it can be formalized as follows:

$$(ROS \vee FOXO1) \wedge\neg(mTORC1 \vee mTORC2)$$

**EOMES:**

In the same way as other signature transcriptional factors, like GATA3, TBET and RORγT, EOMES is able to create a positive feedback loop to maintain its own gene expression (17). However, there are other factors that increase the expression of EOMES, such as the activity of FOXO1 (7) and the signaling of type I IFN (38). Indirectly, ROS also activates EOMES, since ROS activates FOXO1(37). Nevertheless, mTORC2 signaling inhibits EOMES (39). Collectively, this information may be represented in logic terms as follows:

$$(ROS \vee EOMES \vee IFNI \vee FOXO1 ) \wedge\neg(mTORC2 )$$

**mTORC1:**

The master regulator mTORC1 can be activated by amino-acids (9), by cytokines signaling such as IL-12 (7) or by TCR activation (40). On the contrary, mTORC1 can be repressed by PD-1 (41) or by mTORC2 (42). Interestingly, cytokines such as IL-15 may activate a series of downstream inhibitory effects. Firstly, IL-15 activates HIF-1 (43), which induce the expression of REED1(44) and this protein is a potent inhibitor of mTORC1 (45). Therefore, we can represent this information as follows:

$$\left( aa \vee ROS\vee IL12\vee IL12s \vee Akt \right)\wedge\neg\left( mTORC2 \vee PD1 \vee IL15s \right)$$

**mTORC2:**

Regarding the other master regulator, mTORC2, it has been reported that growth factors are responsible for its activation (46). In the same way, ROS activates mTORC2 (47) but the activity of mTORC1 might inhibit mTORC2 through the activity of S6K (48). In other words:

$$(GFs \vee ROS) \wedge\neg mTORC1$$

**ROS:**

Experimental evidence showed that glucose (41), free fat acids (41), ceramides (49), and ethanol (50) increases the ROS production. However, SOD destroys ROS (51). Therefore, we can express these data as follows:

$$Glucose \vee FFAs \vee Ceramide \vee EtOH) \wedge\neg SOD$$

**AKT:**

Concerting AKT regulation, it has been documented that cytokines such as IL-4 (52), IFN-γ (53), and IL-10 (54) as well as mTORC2 (48) can activate AKT . This information might be rewritten as follows:

$$(IFN\gamma\vee IL4 \vee IL10 \vee mTORC2)$$

**GLUT1:**

It has been reported that AKT directly activates GLUT1(40). At transcriptional level, EOMES is required to sustain effector functions of CD8+ T cells, including the expression of GLUT1(55). Nevertheless, other transcription factors such as FOXO1 might induce the expression of miR-378 (56), which inhibits the expression of GLUT1 (57). In other words:

$$Akt \wedge EOMES \wedge\neg FOXO1$$

**Granzyme B:**

The cytotoxic protein Granzyme B is directly activated by T-BET (58), which means that all factors that inhibit T-BET downregulate Granzyme B, such as GATA3, FOXP3 and RORγT. Interestingly, it has been reported that FOXO1 inhibits the expression of Granzyme B (7). Collectively, this information can be represented as follows:

$$TBET \wedge\neg(GATA3\vee ROR\gamma T\vee FOXP3\vee FOXO1)$$

**SOD:**

Experimental observations showed that FOXO1 increases the expression of SOD (59), which can be induced with high levels of ROS (51). However, Ceramide inhibits SOD activation (49). Then, this can be expressed as follows:

$$ROS \wedge(FOXO1) \wedge\neg(Ceramide )$$

**BCL-2**:

Concerning BCL-2, it has been reported that FOXO1 activates BCL2 (60), while ROS represses BCL-2 activity by targeting ERK (61). In other words:

$$(FOXO1) \wedge\neg ROS$$

**Caspase-3:**

Finally, the last node is Caspase-3. This enzyme is a self-activating enzyme (62) that once it is activated, there is no way to stop it. FasL is an external signal that triggers Caspase-3 activation (63). In the same way, an internal signal that might trigger Caspase-3 is high levels of ROS (49). On the contrary, BCL-2 protects against BCL-2 activation (64). Thus:

$$FasL \wedge(ROS \vee Casp3) \wedge\neg BCL2$$

## 2. Biological bases of labeling rules

In order to deal with all attractors of the models, we used labeling rules to identify each attractor according to its genotype with a particular phenotype or cell state. We used transcriptional signatures to construct these labeling rules. In the following section we describe the biological bases of each labeling rule.

**Naïve:**

In general, experimentally it has been observed that naïve CD8+ T cells lack of EOMES and present a stable expression of FOXO1 (65). Also, it is expected that other transcriptional signatures such as GATA3, T-BET, RORγT and FOXP3 were absent (66).

$$\neg(EOMES \vee TBET \vee GATA3 \vee ROR\gamma T \vee FOXP3)$$

**Effector:**

On the other hand, it has been reported that effector CD8+ T cells present high levels of EOMES and low levels of FOXO1(65). In the same way, it is expected that lymphocytes present one transcriptional signature such as T-BET, GATA3, RORγT or FOXP3 (66).

$$\neg FOXO1 \wedge EOMES \wedge( TBET \vee GATA3 \vee ROR\gamma T \vee FOXP3)$$

**Memory:**

Regarding memory CD8+ T cells, it has been noted that EOMES and FOXO1 are equally expressed in such lymphocytes (65). Also, it has been proposed that memory cells may preserve their transcriptional signatures, as it has been reported in Tc17 cells (67). Thus, we can classify attractors with the following formalism:

$$FOXO1 \wedge EOMES \wedge( TBET \vee GATA3 \vee ROR\gamma T \vee FOXP3)$$

**Tc0:**

This phenotype does not present any transcriptional signature such as T-BET, GATA3, RORγT or FOXP3. This phenotype has no effector functions, thus it lacks EOMES.

$$\neg(EOMES \vee TBET \vee GATA3 \vee ROR\gamma T \vee FOXP3)$$

**Tc1:**

This phenotype is activated by the presence of IL-12 and IFN-γ (68), which activates T-BET and IFN-γ. This phenotype is an effector one, which implies that EOMES must be expressed while FOXO1 is repressed. Furthermore, this phenotype inhibits other transcriptional signatures such as GATA3, RORγT or FOXP3.

$$EOMES \wedge(TBET \wedge IFN\gamma) \wedge\neg FOXO1 \wedge\neg(GATA3 \vee ROR\gamma T \vee FOXP3)$$

**Tc2:**

This phenotype is activated by the presence of IL-4 (69), which activates GATA3 and IL-4. This phenotype is an effector one, which means that EOMES must be expressed while FOXO1 is repressed. Furthermore, this phenotype inhibits other transcriptional signatures such as T-BET, RORγT or FOXP3.

$$EOMES \wedge(GATA3 \wedge IL4) \wedge\neg FOXO1 \wedge\neg(TBET \vee ROR\gamma T \vee FOXP3)$$

**Tc17:**

This phenotype is differentiated by IL-6 and TGF-β (68) , which activates RORγT as well as IL-17 production. Similarly, to Tc1 and Tc2, this is an effector phenotype in which EOMES must be expressed while FOXO1 is repressed. Moreover, this phenotype represses other transcription factors such as T-BET, GATA3 or FOXP3.

$$EOMES \wedge(ROR\gamma T) \wedge\neg FOXO1 \wedge\neg(TBET \vee GATA3 \vee FOXP3)$$

**TcReg**:

This regulatory phenotype is activated by IL-12 and IL-4 (27). In this cells, FOXP3 can be activated (70) as well as IL-10 (71). This effector phenotype presents high levels of EOMES, low levels of FOXO1 and inhibits T-BET, GATA3 or RORγT:

$$EOMES \wedge(FOXP3 \wedge IL10) \wedge\neg FOXO1 \wedge\neg(TBET \vee GATA3 \vee ROR\gamma T)$$

## 3. Algorithm to reduce networks

The method we used to reduce the network consists of looking for the interactions that lead to highly connected nodes with non-linearities, such as positive or negative feedback loops. Then, all the nodes that arrive in a linear way to said regulation nodes were considered as a linear chain of variables related to each other in the following form:

$$x_{1}\to x_{2}\to x_{3}\to x_{4}\to\ldots\to x_{n}$$

Applying the transitivity property, we can say that:

$$x_{1}\to x_{n}$$

This method was developed, validated and verified by Villarreal and colleagues in 2012 (72), and is similar to the method developed by Naldi and colleagues in 2009 (73), where the intermediate nodes are removed (only if not self-regulated) from the original network and direct interactions are drawn from the regulators to the targets of the removed node, along with adaptations in the logical rules of the target nodes. To briefly exemplify how the method works, let us look at the activation of GATA3: To activate the transcription of GATA3, it is simultaneously required the presence of active homodimers of STAT5/STAT5 and STAT6/STAT6 (represented as $STAT5^{*}$ and $STAT6^{*}$), which implies that both cytokines, IL-2 and IL-4, must interact with their receptors and these with a series of downstream proteins to induce GATA3 activation. In other words:

$$IL2\to IL2R\to JAK2\to STAT5\to STAT5^{*}\to GATA3$$

$$IL4\to IL4R\to JAK1\to STAT6\to STAT6^{*}\to GATA3$$

These sequences of interactions can be logically represented as follows:

$$IL2\left( t+1 \right)=IL2\left( t \right)$$

$$IL2R\left( t+1 \right)=IL2\left( t \right)$$

$$JAK2\left( t+1 \right)=IL2R\left( t \right)$$

$$STAT5\left( t+1 \right)=JAK2\left( t \right)$$

$$STAT5^{*}\left( t+1 \right)=STAT5\left( t \right)$$

$$IL4\left( t+1 \right)=IL4\left( t \right)$$

$$IL4R\left( t+1 \right)=IL4\left( t \right)$$

$$JAK1\left( t+1 \right)=IL4R\left( t \right)$$

$$STAT6\left( t+1 \right)=JAK1\left( t \right)$$

$$STAT6^{*}\left( t+1 \right)=STAT6\left( t \right)$$

$$GATA3\left( t+1 \right)=STAT6^{*}\left( t \right) \wedge STAT5^{*}\left( t \right)$$

Applying transitivity to interactions, we can simplify them as follows:

$$IL2\to GATA3$$

$$IL4\to GATA3$$

In other words:

$$GATA3\left( t+1 \right)=IL4\left( t \right) \wedge IL2\left( t \right)$$

The same reasoning was applied to all nodes of the GRN.

## 4. Stability test of phenotypes

To assess the stability of network phenotypes in the face of stochastic variations, we first assigned a noise value $\delta$ (in this case, 30%). Then, for each time step, we generated a random number between 0 and 1 ($p$), and if that number was less than or equal to the noise level, then the logic rule "disobeys" its normal behavior, giving as a result the value complementary to what should normally happen. In other words:

$$if p\leq\delta, then: f_{i}=0\to f_{i}=1$$

We implemented this random perturbation for all nodes of the network, to understand how robust the phenotypes of CD8+ T cells are. Finally, we calculated attractors produced by random fluctuations with their respective basins of attraction using C#. As a result of this procedure (Supplementary Figure 6), we found that Tc0 was the less stable phenotype since its frequency has been reduced in 29.3%. On the other hand, TcReg phenotype was the most stable of all, reduced only in a 0.5% of its frequency (Supplementary Figure 6). Other phenotypes were reduced in 24.2% for Tc1, 11.6% for Tc2 and 8.9% for Tc17. This method allowed us to prove that the model is robust enough to maintain its behavior in presence of random fluctuations.

## 5. Supplementary Tables

**Supplementary Table 1**. Logic rules with their biologic references*

| **Node** | **Logic rule** | **References** |
| --- | --- | --- |
| **T-BET** | $\left( \left( IFN\gamma\vee\left( IL12s \wedge\neg\left( IL6s \vee IL4 \vee IL10 \right) \right) \right) \vee TBET \right)\wedge\neg\left( IL4 \vee GATA3 \vee IL6s \vee FOXO1 \right)$ | (1 – 7) |
| **IFN-γ** | $(IFN\gamma s \vee IFNI \vee((IFN\gamma\vee TBET \vee EOMES) \wedge mTORC1 \wedge\neg(GATA3 \vee TGF\beta))) \wedge\neg(IL6s \vee IL4 \vee IL10)$ | (3, 6, 8 – 14) |
| **GATA3** | $\left( \left( IL2s \wedge IL4 \right)\vee EOMES \vee GATA3 \right)\wedge\neg\left( TBET \vee TGF\beta\vee IL6s \vee IFN\gamma\right)$ | (3, 6, 15 – 17) |
| **IL-4** | $(IL4s \vee(GATA3 \wedge(IL2s \vee IL4) \wedge\neg TBET)) \wedge\neg(IFN\gamma\vee IL6s )$ | (6, 11, 18, 19) |
| **RORγT** | $(IL6s \wedge TGF\beta) \wedge\neg(TBET \vee FOXP3 \vee GATA3 \vee FOXO1)$ | (20 – 24) |
| **IL-10** | $(IL10s \vee EOMES \vee(IL10 \wedge(IFN\gamma\vee IL6s \vee TGF\beta\vee GATA3 ))) \wedge mTORC1$ | (6, 25 – 32) |
| **FOXP3** | $\left( \left( IL2s \vee IL12s \right)\wedge\left( TGF\beta\vee FOXP3 \vee IL4 \vee\left( FOXO1 \right) \right) \right)\wedge\neg\left( IL6s \vee ROR\gamma T \right)$ | (20, 26, 33 – 35) |
| **FOXO1** | $\left( ROS \vee FOXO1 \right)\wedge\neg\left( mTORC1 \vee mTORC2 \right)$ | (7, 36, 37) |
| **EOMES** | $\left( ROS \vee EOMES \vee IFNI \vee FOXO1 \right)\wedge\neg\left( mTORC2 \right)$ | (7, 17, 37 – 39) |
| **mTORC1** | $\left( aa \vee ROS\vee IL12\vee IL12s \vee Akt \right)\wedge\neg\left( mTORC2 \vee PD1 \vee IL15s \right)$ | (7, 9, 41 – 45) |
| **mTORC2** | $\left( GFs \vee ROS \right)\wedge\neg mTORC1$ | (46 – 47) |
| **ROS** | $Glucose \vee FFAs \vee Ceramide \vee EtOH) \wedge\neg SOD$ | (41, 49 – 51) |
| **AKT** | $\left( IFN\gamma\vee IL4 \vee IL10 \vee mTORC2 \right)$ | (42, 52 – 54) |
| **GLUT1** | $Akt \wedge EOMES \wedge\neg FOXO1$ | (40, 55 – 57) |
| **GRZB** | $TBET \wedge\neg\left( GATA3\vee ROR\gamma T\vee FOXP3\vee FOXO1 \right)$ | (7, 58) |
| **SOD** | $ROS \wedge\left( FOXO1 \right)\wedge\neg\left( Ceramide \right)$ | (49, 51, 59) |
| **BCL2** | $\left( FOXO1 \right)\wedge\neg ROS$ | (60, 61) |
| **CASP3** | $FasL \wedge\left( ROS \vee Casp3 \right)\wedge\neg BCL2$ | (62 – 64) |

*In this table we used $\wedge$ to represent the logic operator “AND”, $\vee$ to represent “OR” and finally “$\neg$” to represent “NOT”.

**Supplementary Table 2**. Update times for the asynchronous implementation

| **Node** | **Update time (time steps)** | **References*** |
| --- | --- | --- |
| T-BET | $1/8$ | (74) |
| IFN-γ | $1/4$ | (75) |
| GATA3 | $1/8$ | (76) |
| IL-4 | $1/4$ | (75) |
| RORγT | $1/4$ | (22) |
| IL-10 | $1/4$ | (75) |
| FOXP3 | $1/4$ | (77) |
| FOXO1 | $1/4$ | (78) |
| EOMES | $1/4$ | (79) |
| mTORC1 | $1/2$ | (80) |
| mTORC2 | $1/2$ | (80) |
| ROS | $1$ | (81) |
| Akt | $1/2$ | (82) |
| GLUT1 | $1/2$ | (83) |
| Granzyme B | $1/8$ | (84) |
| SOD | $1/2$ | (85) |
| BCL-2 | $1/4$ | (86) |
| Caspase-3 | $1/4$ | (86) |

*To implement this model with an asynchronous update scheme, we assigned 8 time-steps to T-BET, GATA3 and Granzyme B because T-BET reaches its maximal peak after 8 hours post-induction (74), on the other hand, GATA3 presents a similar time to reach its maximum expression (76). In the same way, Granzyme B reaches its maximum when T-BET does (84). On the other hand, we assigned 4 time-steps to IFN-γ (75), IL-4 (75), RORγT (22), IL-10 (75), FOXP3 (77), FOXO1 (78), EOMES (79), BCL2 and Caspase-3 (86) because the mRNA synthesis of such genes occurs in a similar temporal window, 4 hours after gene stimulation. Similarly, we assigned 2 time-steps to SOD, mTORC1, mTORC2 (80), AKT (82) and GLUT1 (83), since its activation do not depend on transcriptional activation, instead, they are enzymatically activated by their regulators, which takes around 2 hours. Finally, we assigned 1 time-step to ROS activation, since ROS needs almost 1 hour to reach high levels (81).

## 6. Supplementary Figures


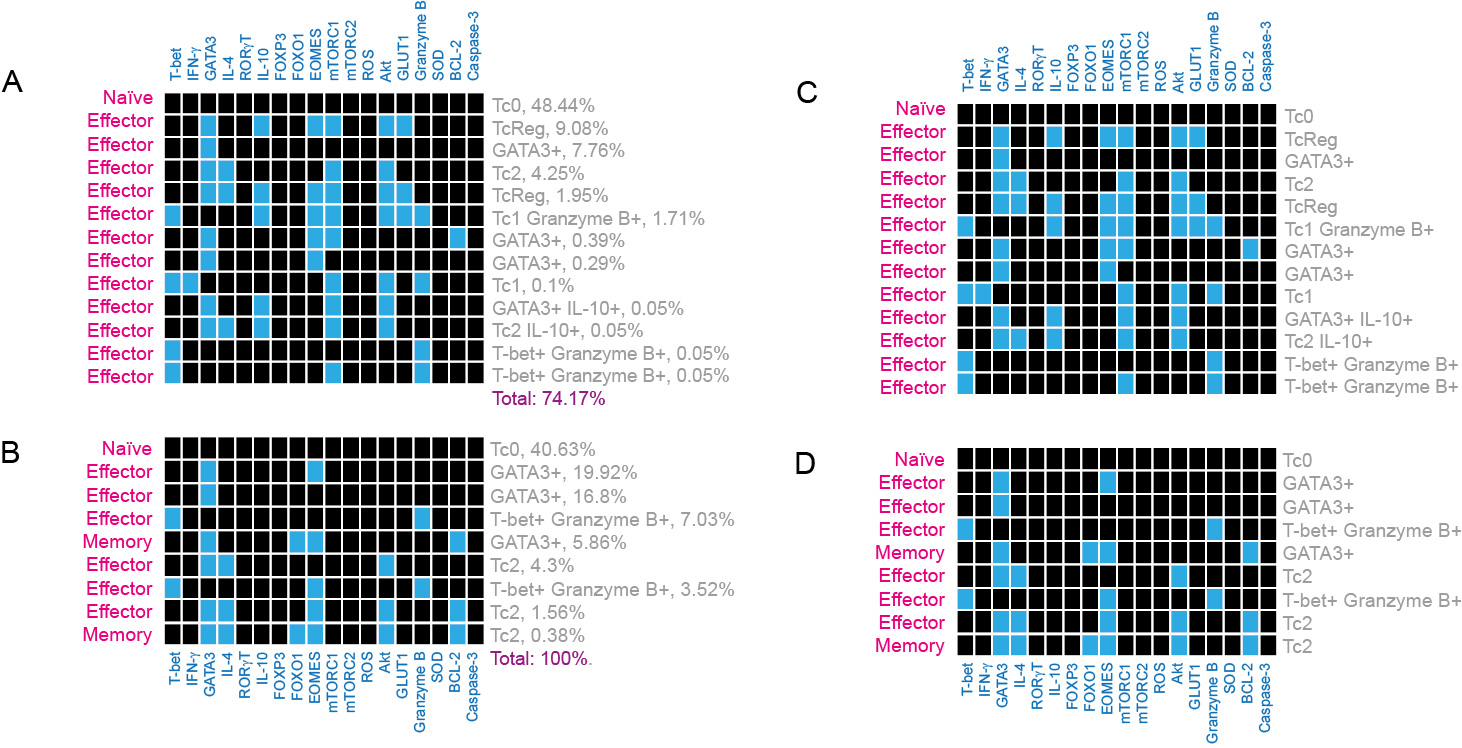


**Supplementary Figure 1.** Attractors found in Tc0 conditions as identified by our method and validated using GINSIM. (A) Attractors found in absence of IL-15, (B) attractors found in presence of IL-15. (C) Attractors found in absence of IL-15 using GINSIM, (D) attractors found in presence of IL-15 using GINSIM. In this figure, black squares represent “inactive state” (i.e., 0) and blue squares are used to represent “active state” (or 1). We show the frequency ($f_{k}$) of each attractor found with our algorithm in panels A and B, and we present the sum of all frequencies in purple as percentages. Pink labels are used to describe whether the attractor found corresponds either to naïve cells, effector cells or memory cell. Gray labels describe molecular measurable features of each attractor, while blue labels identify each node of the reduced network. In supplementary figures 1-5, only attractors of the type “stable state” are considered.


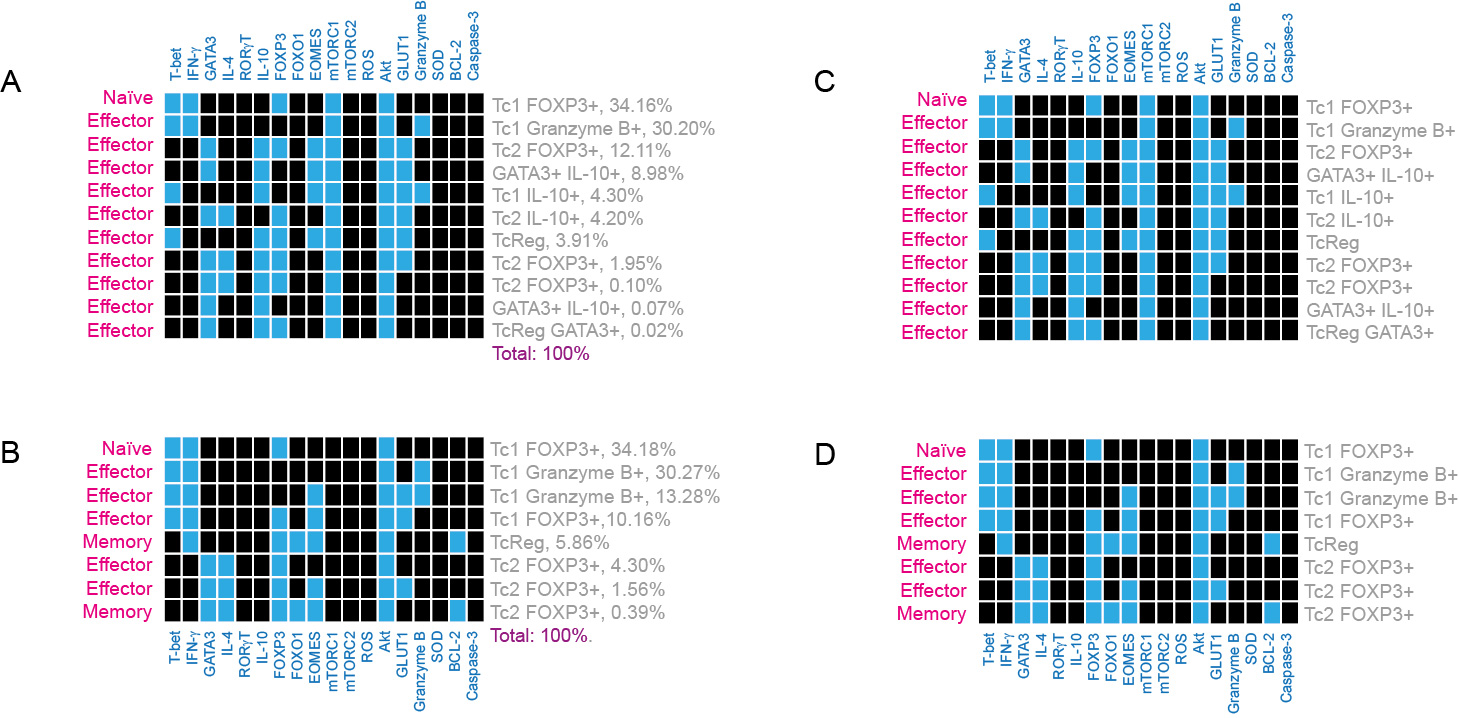


**Supplementary Figure 2.** Attractors found after TCR stimulation in Tc1 conditions (IL-12 and IFN-γ) as identified by our method and validated using GINSIM. (A) Attractors found in absence of IL-15, (B) attractors found in presence of IL-15. (C) Attractors found in absence of IL-15 with GINSIM, (D) attractors found in presence of IL-15 with GINSIM. In this figure, black squares represent “inactive state” (i.e., 0) and blue squares are used to represent “active state” (or 1). We show the frequency ($f_{k}$) of each attractor found with our algorithm in panels A and B, and we present the sum of all frequencies in purple as percentages. Pink labels are used to describe whether the attractor found corresponds either to naïve cells, effector cells or memory cell. Gray labels describe molecular measurable features of each attractor, while blue labels identify each node of the reduced network.


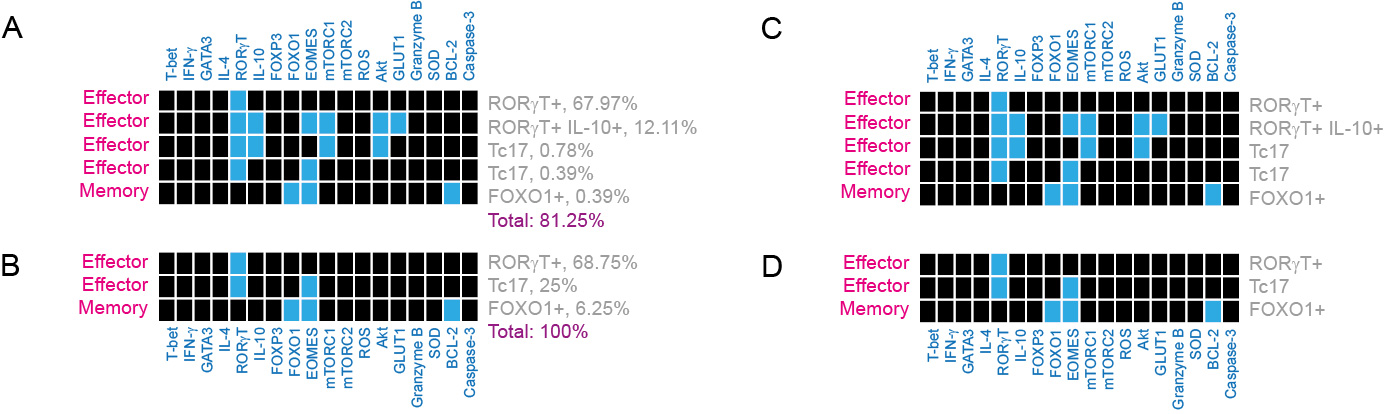


**Supplementary Figure 3.** Attractors found after TCR stimulation in Tc17 conditions (IL-6 and TGF-β) as identified by our method and validated using GINSIM. (A) Attractors found in absence of IL-15, (B) attractors found in presence of IL-15. (C) Attractors found in absence of IL-15 with GINSIM, (D) attractors found in presence of IL-15 with GINSIM. In this figure black squares represent “inactive state” (i.e., 0) and blue squares are used to represent “active state” (or 1). We show the frequency ($f_{k}$) of each attractor found with our algorithm in panels A and B, and we present the sum of all frequencies in purple as percentages. Pink labels are used to describe whether the attractor found corresponds either to naïve cells, effector cells or memory cell. Gray labels describe molecular measurable features of each attractor, while blue labels identify each node of the reduced network.


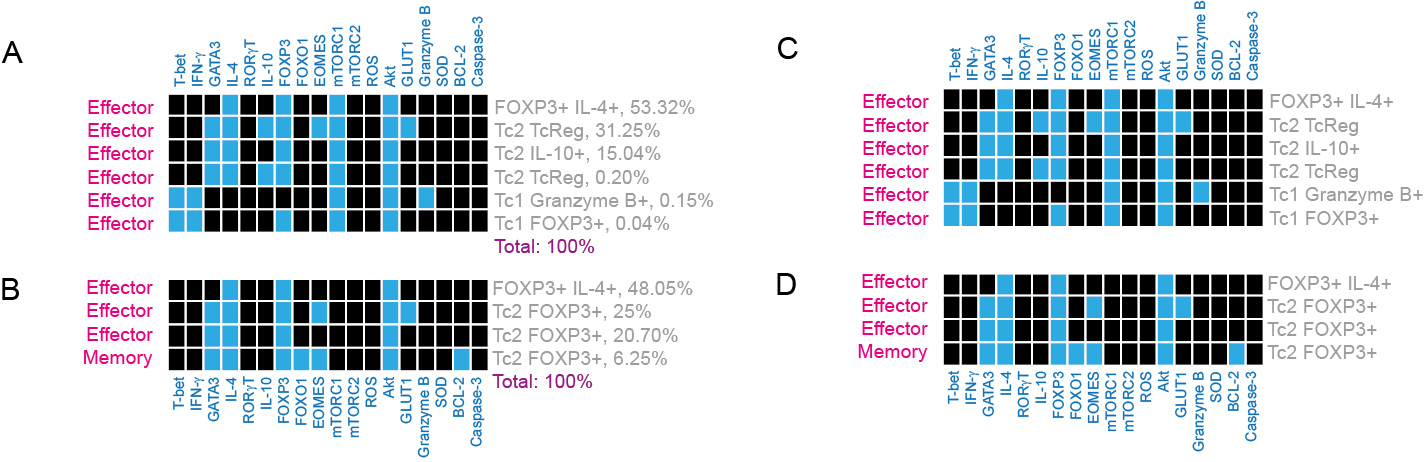


**Supplementary Figure 4.** Attractors found after TCR stimulation in TcReg conditions (IL-12 and IL-4) as identified by our method and validated using GINSIM. (A) Attractors found in absence of IL-15, (B) attractors found in presence of IL-15. (C) Attractors found in absence of IL-15 with GINSIM, (D) attractors found in presence of IL-15 with GINSIM. In this figure, black squares represent “inactive state” (i.e., 0) and blue squares are used to represent “active state” (or 1). We show the frequency ($f_{k}$) of each attractor found with our algorithm in panels A and B, and we present the sum of all frequencies in purple as percentages. Pink labels are used to describe whether the attractor found corresponds either to naïve cells, effector cells or memory cell. Gray labels describe molecular measurable features of each attractor, while blue labels identify each node of the reduced network.


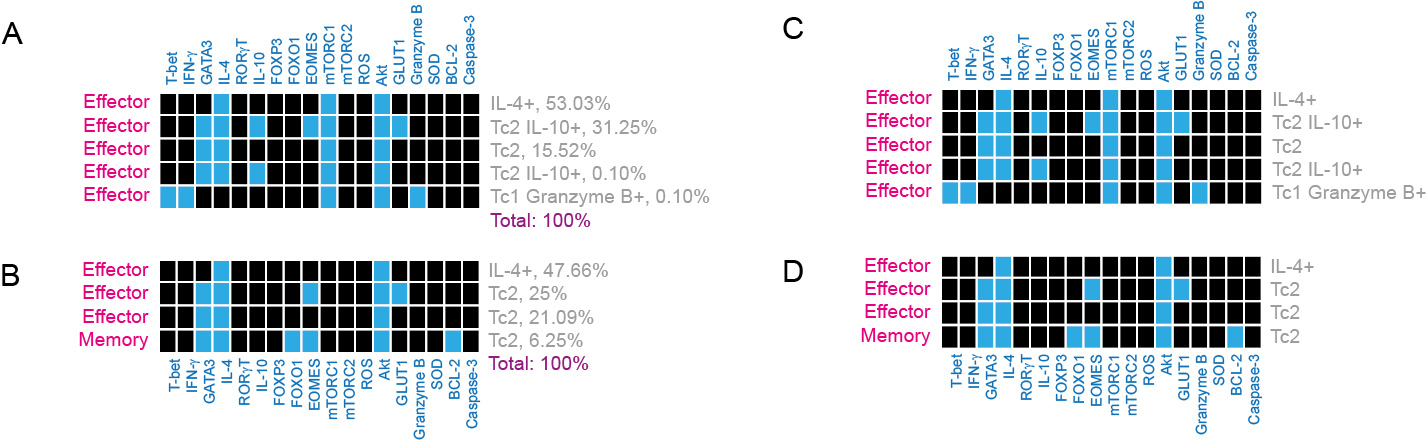


**Supplementary Figure 5.** Attractors found after TCR stimulation in Tc2 conditions (IL-4) as identified by our method and validated using GINSIM. (A) Attractors found in absence of IL-15, (B) attractors found in presence of IL-15. (C) Attractors found in absence of IL-15 with GINSIM, (D) attractors found in presence of IL-15 with GINSIM. In this figure, black squares represent “inactive state” (i.e., 0) and blue squares are used to represent “active state” (or 1). We show the frequency ($f_{k}$) of each attractor found with our algorithm in panels A and B, and we present the sum of all frequencies in purple as percentages. Pink labels are used to describe whether the attractor found corresponds either to naïve cells, effector cells or memory cell. Gray labels describe molecular measurable features of each attractor, while blue labels identify each node of the reduced network.


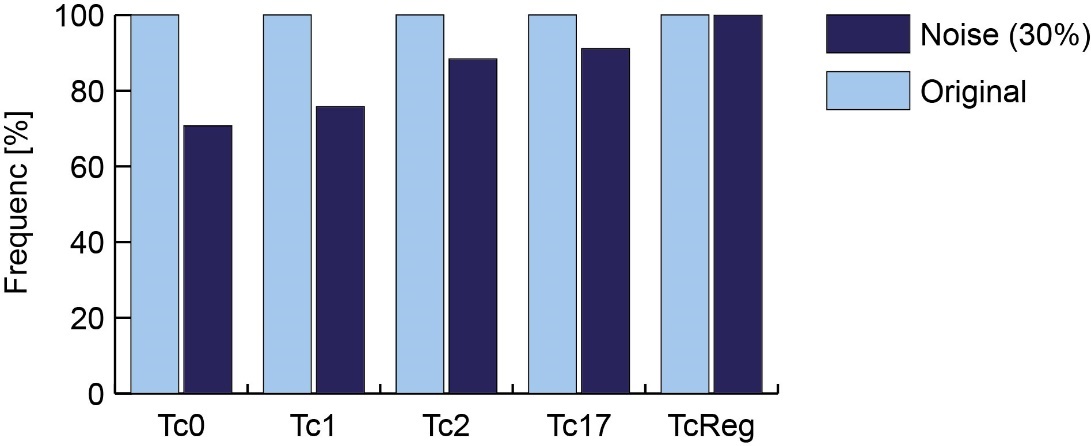


**Supplementary Figure 6.** In this figure, we present the outcome of the sensitivity analysis performed to the GRN. Here, the normalized frequency of each CD8+ T cell phenotypes decreased as a result of preforming random variations on each node of the network. Tc0 phenotype was the most sensitive of all because its frequency was reduced to 70.7% of its original size. On the other hand, TcReg phenotype was the most robust of all, since its frequency was reduced to 99.9%. These simulations were executed with a 30% of randomness level.

## 7. Supplementary References

1. Ylikoski E, Lund R, Kyläniemi M, Filén S, Kilpeläinen M, Savolainen J, et al. IL-12 up-regulates T-bet independently of IFN-γ in human CD4+ T cells. Eur J Immunol. 2005;

2. Iwata S, Mikami Y, Sun HW, Brooks SR, Jankovic D, Hirahara K, et al. The Transcription Factor T-bet Limits Amplification of Type I IFN Transcriptome and Circuitry in T Helper 1 Cells. Immunity. 2017;

3. Zhu J, Jankovic D, Oler AJ, Wei G, Sharma S, Hu G, et al. The Transcription Factor T-bet Is Induced by Multiple Pathways and Prevents an Endogenous Th2 Cell Program during Th1 Cell Responses. Immunity. 2012;

4. Takeda H, Tanaka T, Shi W, Matsumoto M, Minami M, Kashiwamura SI, et al. Essential role of Stat6 in IL-4 signalling. Nature. 1996;

5. Tamachi T, Takatori H, Fujiwara M, Hirose K, Maezawa Y, Kagami S ichiro, et al. STAT6 inhibits T-bet-independent Th1 cell differentiation. Biochem Biophys Res Commun. 2009;

6. Yoshimura A, Suzuki M, Sakaguchi R, Hanada T, Yasukawa H. SOCS, inflammation, and autoimmunity. Frontiers in Immunology. 2012.

7. Rao RR, Li Q, Bupp MRG, Shrikant PA. Transcription Factor Foxo1 Represses T-bet-Mediated Effector Functions and Promotes Memory CD8 + T Cell Differentiation. Immunity. 2012;

8. Gonzales-van Horn SR, Farrar JD. Interferon at the crossroads of allergy and viral infections. J Leukoc Biol. 2015;

9. Zhang Y, Hu H, Liu W, Yan SM, Li Y, Tan L, et al. Amino acids and RagD potentiate mTORC1 activation in CD8 + T cells to confer antitumor immunity. J Immunother Cancer. 2021;

10. Fukuoka N, Harada M, Nishida A, Ito Y, Shiota H, Kataoka T. Eomesodermin promotes interferon-γ expression and binds to multiple conserved noncoding sequences across the Ifng locus in mouse thymoma cell lines. Genes to Cells. 2016;

11. Li-Weber M, Krammer PH. Regulation of IL4 gene expression by T cells and therapeutic perspectives. Nature Reviews Immunology. 2003.

12. Kaminuma O, Kitamura F, Kitamura N, Miyagishi M, Taira K, Yamamoto K, et al. GATA-3 suppresses IFN-γ promoter activity independently of binding to cis-regulatory elements. FEBS Lett. 2004;

13. Deng Y, Kerdiles Y, Chu J, Yuan S, Wang Y, Chen X, et al. Transcription factor foxo1 is a negative regulator of natural killer cell maturation and function. Immunity. 2015;

14. Park IK, Letterio JJ, Gorham JD. TGF-β1 inhibition of IFN-γ-induced signaling and Th1 gene expression in CD4+ T cells is Smad3 independent but MAP kinase dependent. Mol Immunol. 2007;

15. Hinks TSC, Hoyle RD, Gelfand EW. CD8+ Tc2 cells: Underappreciated contributors to severe asthma. European Respiratory Review. 2019.

16. Wan YY. GATA3: A master of many trades in immune regulation. Trends in Immunology. 2014.

17. Kidder BL, Palmer S. Examination of transcriptional networks reveals an important role for TCFAP2C, SMARCA4, and EOMES in trophoblast stem cell maintenance. Genome Res. 2010;

18. Kaplan MH, Wurster AL, Smiley ST, Grusby MJ. Stat6-dependent and -independent pathways for IL-4 production. J Immunol. 1999;

19. Zhuang Y, Huang Z, Nishida J, Brown M, Zhang L, Huang H. A continuous T-bet expression is required to silence the interleukin-4-producing potential in T helper type 1 cells. Immunology. 2009;

20. Tanaka T, Narazaki M, Kishimoto T. Anti-interleukin-6 receptor antibody, tocilizumab, for the treatment of autoimmune diseases. FEBS Letters. 2011.

21. Lainé A, Martin B, Luka M, Mir L, Auffray C, Lucas B, et al. Foxo1 Is a T Cell–Intrinsic Inhibitor of the RORγt-Th17 Program. J Immunol. 2015;

22. Ichiyama K, Yoshida H, Wakabayashi Y, Chinen T, Saeki K, Nakaya M, et al. Foxp3 inhibits RORγt-mediated IL-17A mRNA transcription through direct interaction with RORγt. J Biol Chem. 2008;

23. Lee JS, Cua DJ. The emerging landscape of RORγt biology. Immunity. 2014.

24. Lazarevic V, Chen X, Shim J-H, Hwang E-S, Jang E, Bolm AN, et al. Transcription factor T-bet represses Th17 differentiation by preventing Runx1-mediated activation of the RORγt gene. Nat Immunol. 2011;

25. Brooks DG, Walsh KB, Elsaesser H, Oldstone MBA. IL-10 directly suppresses CD4 but not CD8 T cell effector and memory responses following acute viral infection. Proc Natl Acad Sci U S A. 2010;

26. Vieyra-Lobato MR, Vela-Ojeda J, Montiel-Cervantes L, López-Santiago R, Moreno-Lafont MC. Description of CD8+ regulatory T lymphocytes and their specific intervention in Graft-versus-Host and infectious diseases, autoimmunity, and cancer. Journal of Immunology Research. 2018.

27. Noble A, Giorgini A, Leggat JA. Cytokine-induced IL-10-secreting CD8 T cells represent a phenotypically distinct suppressor T-cell lineage. Blood. 2006;

28. Hedrich CM, Rauen T, Apostolidis SA, Grammatikos AP, Rodriguez NR, Ioannidis C, et al. Stat3 promotes IL-10 expression in lupus T cells through trans-activation and chromatin remodeling. Proc Natl Acad Sci U S A. 2014;

29. Huss DJ, Winger RC, Peng H, Yang Y, Racke MK, Lovett-Racke AE. TGF-β Enhances Effector Th1 Cell Activation but Promotes Self-Regulation via IL-10. J Immunol. 2010;

30. Reiser J, Sadashivaiah K, Furusawa A, Banerjee A, Singh N. Eomesodermin driven IL-10 production in effector CD8 + T cells promotes a memory phenotype. Cell Immunol. 2019;

31. Shoemaker J, Saraiva M, O’Garra A. GATA-3 Directly Remodels the IL-10 Locus Independently of IL-4 in CD4 + T Cells . J Immunol. 2006;

32. Jung U, Foley JE, Erdmann AA, Toda Y, Borenstein T, Mariotti J, et al. Ex Vivo Rapamycin Generates Th1/Tc1 or Th2/Tc2 Effector T Cells With Enhanced In Vivo Function and Differential Sensitivity to Post-transplant Rapamycin Therapy. Biol Blood Marrow Transplant. 2006;

33. Zorn E, Nelson EA, Mohseni M, Porcheray F, Kim H, Litsa D, et al. IL-2 regulates FOXP3 expression in human CD4+CD25+ regulatory T cells through a STAT-dependent mechanism and induces the expansion of these cells in vivo. Blood. 2006;

34. Chakraborty S, Panda AK, Bose S, Roy D, Kajal K, Guha D, et al. Transcriptional regulation of FOXP3 requires integrated activation of both promoter and CNS regions in tumor-induced CD8+ Treg cells. Sci Rep. 2017;

35. Ono M. Control of regulatory T-cell differentiation and function by T-cell receptor signalling and Foxp3 transcription factor complexes. Immunology. 2020.

36. Xiong S, Salazar G, Patrushev N, Alexander RW. FoxO1 mediates an autofeedback loop regulating SIRT1 expression. J Biol Chem. 2011;

37. Storz P. Forkhead homeobox type O transcription factors in the responses to oxidative stress. Antioxidants and Redox Signaling. 2011.

38. Martinet V, Tonon S, Torres D, Azouz A, Nguyen M, Kohler A, et al. Type i interferons regulate eomesodermin expression and the development of unconventional memory CD8 + T cells. Nat Commun. 2015;

39. Wang H, Xiao Y, Su L, Cui N, Liu D. MTOR modulates CD8+ T cell differentiation in mice with invasive pulmonary aspergillosis. Open Life Sci. 2018;

40. Salmond RJ. mTOR Regulation of Glycolytic Metabolism in T Cells. 2018;6(September):1–9.

41. Lim AR, Rathmell WK, Rathmell JC. The tumor microenvironment as a metabolic barrier to effector T cells and immunotherapy. Elife. 2020;

42. Lu Z, Shi X, Gong F, Li S, Wang Y, Ren Y, et al. RICTOR/mTORC2 affects tumorigenesis and therapeutic efficacy of mTOR inhibitors in esophageal squamous cell carcinoma. Acta Pharm Sin B. 2020;

43. Coulibaly A, Velásquez SY, Kassner N, Schulte J, Barbarossa MV, Lindner HA. STAT3 governs the HIF-1α response in IL-15 primed human NK cells. Sci Rep. 2021;

44. Lipina C, Hundal HS. Is REDD1 a Metabolic Éminence Grise? Trends in Endocrinology and Metabolism. 2016.

45. Wouters BG, Koritzinsky M. Hypoxia signalling through mTOR and the unfolded protein response in cancer. Nature Reviews Cancer. 2008.

46. Gu Y, Albuquerque CP, Braas D, Zhang W, Villa GR, Bi J, et al. mTORC2 Regulates Amino Acid Metabolism in Cancer by Phosphorylation of the Cystine-Glutamate Antiporter xCT. Mol Cell. 2017;

47. Luo Y, Xu W, Li G, Cui W. Weighing in on mTOR complex 2 signaling: The expanding role in cell metabolism. Oxidative Medicine and Cellular Longevity. 2018.

48. Yang G, Murashige DS, Humphrey SJ, James DE. A Positive Feedback Loop between Akt and mTORC2 via SIN1 Phosphorylation. Cell Rep. 2015;

49. Chang YC, Fong Y, Tsai EM, Chang YG, Chou HL, Wu CY, et al. Exogenous C8-ceramide induces apoptosis by overproduction of ROS and the switch of superoxide dismutases SOD1 to SOD2 in human lung cancer cells. Int J Mol Sci. 2018;

50. Hoek JB, Cahill A, Pastorino JG. Alcohol and mitochondria: A dysfunctional relationship. Gastroenterology. 2002;

51. Petrache I, Medler TR, Richter AT, Kamocki K, Chukwueke U, Zhen L, et al. Superoxide dismutase protects against apoptosis and alveolar enlargement induced by ceramide. Am J Physiol - Lung Cell Mol Physiol. 2008;

52. Rückerl D, Jenkins SJ, Laqtom NN, Gallagher IJ, Sutherland TE, Duncan S, et al. Induction of IL-4Rα-dependent microRNAs identifies PI3K/Akt signaling as essential for IL-4-driven murine macrophage proliferation in vivo. Blood. 2012;

53. Jorgovanovic D, Song M, Wang L, Zhang Y. Roles of IFN-γin tumor progression and regression: A review. Biomarker Research. 2020.

54. Zhu YP, Brown JR, Sag D, Zhang L, Suttles J. Adenosine 5′-Monophosphate–Activated Protein Kinase Regulates IL-10–Mediated Anti-Inflammatory Signaling Pathways in Macrophages. J Immunol. 2015;

55. Wells AC, Daniels KA, Angelou CC, Fagerberg E, Burnside AS, Markstein M, et al. Modulation of let-7 miRNAS controls the differentiation of effector CD8 T cells. Elife. 2017;

56. Cheng Z. The FoxO–Autophagy Axis in Health and Disease. Trends in Endocrinology and Metabolism. 2019.

57. Cannistraci A, Hascoet P, Ali A, Mundra P, Clarke NW, Pavet V, et al. MiR-378a inhibits glucose metabolism by suppressing GLUT1 in prostate cancer. Oncogene. 2022;41(10):1445–55.

58. Lazarevic V, Glimcher LH, Lord GM. T-bet: a bridge between innate and adaptive immunity. Nat Rev Immunol [Internet]. 2013 Nov [cited 2014 May 3];13(11):777–89. Available from: http://www.ncbi.nlm.nih.gov/pubmed/24113868

59. Klotz LO, Sánchez-Ramos C, Prieto-Arroyo I, Urbánek P, Steinbrenner H, Monsalve M. Redox regulation of FoxO transcription factors. Redox Biology. 2015.

60. Delpoux A, Michelini RH, Verma S, Lai CY, Omilusik KD, Utzschneider DT, et al. Continuous activity of Foxo1 is required to prevent anergy and maintain the memory state of CD8 + T cells. J Exp Med. 2018;

61. Guo C, Yang M, Jing L, Wang J, Yu Y, Li Y, et al. Amorphous silica nanoparticles trigger vascular endothelial cell injury through apoptosis and autophagy via reactive oxygen species-mediated MAPK/Bcl-2 and PI3K/Akt/mTORsignaling. Int J Nanomedicine. 2016;

62. Ferreira KS, Kreutz C, Macnelly S, Neubert K, Haber A, Bogyo M, et al. Caspase-3 feeds back on caspase-8, Bid and XIAP in type I Fas signaling in primary mouse hepatocytes. Apoptosis. 2012 May;17(5):503–15.

63. Xue C, Lan-lan W, Bei C, Jie C, Wei-hua F. Abnormal Fas/FasL and caspase-3-mediated apoptotic signaling pathways of T lymphocyte subset in patients with systemic lupus erythematosus. Cell Immunol. 2006;

64. Swanton E, Savory P, Cosulich S, Clarke P, Woodman P. Bcl-2 regulates a caspase-3/caspase-2 apoptotic cascade in cytosolic extracts. Oncogene. 1999;

65. Chen Y, Zander R, Khatun A, Schauder DM, Cui W. Transcriptional and Epigenetic Regulation of Effector and Memory CD8 T Cell Differentiation. Vol. 9, Frontiers in Immunology. 2018.

66. Lauvau G, Goriely S. Memory CD8+ T Cells: Orchestrators and Key Players of Innate Immunity? PLoS Pathog. 2016;

67. Flores-Santibáñez F, Cuadra B, Fernández D, Rosemblatt M V., Núñez S, Cruz P, et al. In vitro-generated Tc17 cells present a memory phenotype and serve as a reservoir of Tc1 cells in vivo. Front Immunol. 2018;

68. Kumaresan PR, da Silva TA, Kontoyiannis DP. Methods of controlling invasive fungal infections using CD8+ T cells. Frontiers in Immunology. 2018.

69. Kemp RA, Bäckström BT, Ronchese F. The phenotype of type 1 and type 2 CD8+ T cells activated in vitro is affected by culture conditions and correlates with effector activity. Immunology. 2005;

70. Mishra S. CD8+ Regulatory T Cell – A Mystery to Be Revealed. Frontiers in Immunology. 2021.

71. Noble A, Mehta H, Lovell A, Papaioannou E, Fairbanks L. IL-12 and IL-4 activate a CD39-dependent intrinsic peripheral tolerance mechanism in CD8+ T cells. Eur J Immunol. 2016;

72. Villarreal C, Padilla-Longoria P, Alvarez-Buylla ER. General theory of genotype to phenotype mapping: Derivation of epigenetic landscapes from n-node complex gene regulatory networks. Phys Rev Lett. 2012 Sep;109(11):118102.

73. Naldi A, Remy E, Thieffry D, Chaouiya C. A reduction of logical regulatory graphs preserving essential dynamical properties. Lect Notes Comput Sci. 2009;5688 LNBI:266–80.

74. Placek K, Gasparian S, Coffre M, Maiella S, Sechet E, Bianchi E, et al. Integration of Distinct Intracellular Signaling Pathways at Distal Regulatory Elements Directs T-bet Expression in Human CD4 + T Cells . J Immunol. 2009;183(12):7743–51.

75. Provenzano M, Mocellin S, Bonginelli P, Nagorsen D, Kwon SW, Stroncek D. Ex vivo screening for immunodominant viral epitopes by quantitative real time polymerase chain reaction (qRT-PCR). J Transl Med. 2003;1(May 2014).

76. Peine M, Rausch S, Helmstetter C, Fröhlich A, Hegazy AN, Kühl A a, et al. Stable T-bet(+)GATA-3(+) Th1/Th2 hybrid cells arise in vivo, can develop directly from naive precursors, and limit immunopathologic inflammation. PLoS Biol [Internet]. 2013 Aug [cited 2013 Nov 15];11(8):e1001633. Available from: http://www.pubmedcentral.nih.gov/articlerender.fcgi?artid=3747991&tool=pmcentrez&rendertype=abstract

77. Schmidt A, Marabita F, Kiani NA, Gross CC, Johansson HJ, Éliás S, et al. Time-resolved transcriptome and proteome landscape of human regulatory T cell (Treg) differentiation reveals novel regulators of FOXP3. BMC Biol. 2018;16(1):1–35.

78. Frescas D, Valenti L, Accili D. Nuclear trapping of the forkhead transcription factor FoxO1 via sirt-dependent deacetylation promotes expression of glucogenetic genes. J Biol Chem. 2005;280(21):20589–95.

79. Bourillot PY, Garrett N, Gurdon JB. A changing morphogen gradient is interpreted by continuous transduction flow. Development. 2002;129(9):2167–80.

80. Bauchart-Thevret C, Cui L, Wu G, Burrin DG. Arginine-induced stimulation of protein synthesis and survival in IPEC-J2 cells is mediated by mTOR but not nitric oxide. Am J Physiol - Endocrinol Metab. 2010;299(6).

81. Gerber IB, Dubery IA. Fluorescence microplate assay for the detection of oxidative burst products in tobacco cell suspensions using 2′,7′-dichlorofluorescein. Methods Cell Sci. 2004;25(3–4):115–22.

82. Lee DY, Choi BK, Lee DG, Kim YH, Kim CH, Lee SJ, et al. 4-1BB Signaling Activates the T Cell Factor 1 Effector/β-Catenin Pathway with Delayed Kinetics via ERK Signaling and Delayed PI3K/AKT Activation to Promote the Proliferation of CD8+ T Cells. PLoS One. 2013;8(7).

83. Macintyre AN, Gerriets VA, Nichols AG, Michalek RD, Rudolph MC, Deoliveira D, et al. The glucose transporter Glut1 is selectively essential for CD4 T cell activation and effector function. Cell Metab. 2014;20(1):61–72.

84. Song Z, Zhang T, Li G, Tang Y, Luo Y, Yu G. Tr1 responses are elevated in asymptomatic H. pylori-infected individuals and are functionally impaired in H. pylori-gastric cancer patients. Exp Cell Res. 2018;

85. S. N, K. K, Y. F, M. F, M.I. D, D.L. E, et al. Activation of signal transducer and activator of transcription 3 protects cardiomyocytes from hypoxia/reoxygenation-induced oxidative stress through the upregulation of manganese superoxide dismutase. Circulation. 2001;104(9):979–81.

86. Scheel-Toellner D, Raza K, Assi L, Pilling D, Ross EJ, Lee WY, et al. Differential regulation of nuclear and mitochondrial Bcl-2 in T cell apoptosis. Apoptosis. 2008;13(1):109–17.
