# Supplementary figures and images for: Metabolic alterations impair differentiation and effector functions of CD8+ T cells
Source: Front Immunol. 2022 Aug 2;13:945980. doi: 10.3389/fimmu.2022.945980 (PMC9380903; doi:10.3389/fimmu.2022.945980)

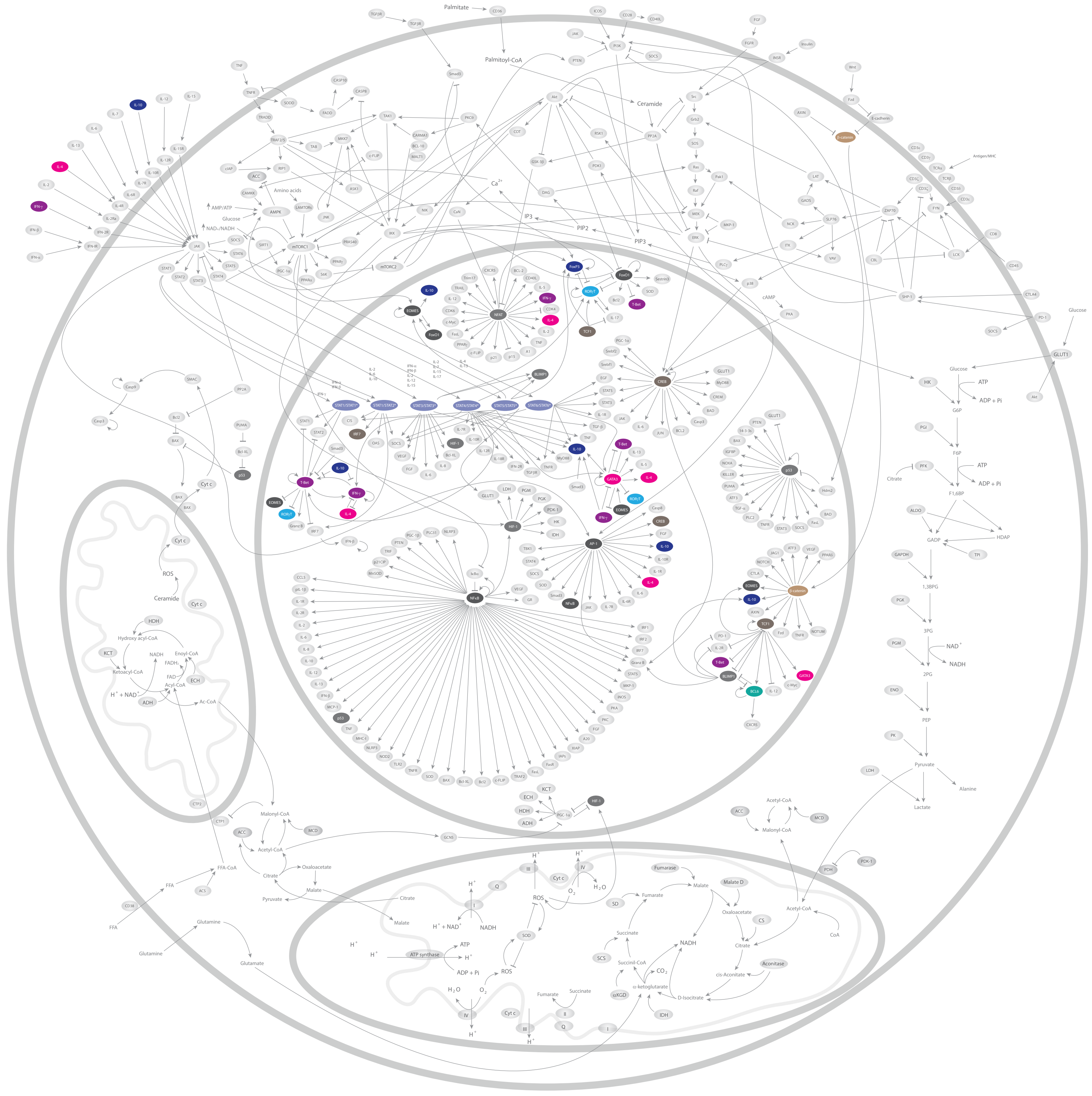

Supplement: Supplementary file 2 [file Image_1.pdf]
